# Mortality in sea lions is associated with the introduction of the H5N1 clade 2.3.4.4b virus in Brazil October 2023: whole genome sequencing and phylogenetic analysis

**DOI:** 10.1186/s12917-024-04137-1

**Published:** 2024-07-02

**Authors:** Andreina de Carvalho Araujo, Andrew Yong Cho, Laura Morais Nascimento Silva, Thais Camilo Corrêa, Gabriela Cristini de Souza, Adriana Silva Albuquerque, Eduardo Macagnan, Cristiane K. M. Kolesnikvoas, Rafael Meurer, Jenyffer Vierheller Vieira, Giulia Gaglianone Lemos, André Silva Barreto, Jeferson Luis Dick, Karina Rejane Groch, Pedro Volkmer de Castilho, Deyvid Amgarten, Fernanda Malta, Michael Miller, Erick G. Dorlass, Soledad Palameta, Sun-Hak Lee, Clarice Weis Arns, Edison L. Durigon, João Renato R. Pinho, Dong-Hun Lee, Helena Lage Ferreira

**Affiliations:** 1 Department of Veterinary Medicine, FZEA- USP, University of São Paulo, 225 Av Duque de Caxias Norte, Pirassununga, SP 13635900 Brazil; 2 Avian Disease Laboratory, College of Veterinary Medicine, Konkuk University, 120 Neungdong-Ro, Gwangjin-Gu, Seoul, 05029 Republic of Korea; 3 Graduate Program in Experimental Epidemiology Applied to Zoonoses, Veterinary Medicine and Animal Science School, University of São Paulo, Av. Prof. Orlando Marques de Paiva, 87, Butantã, São Paulo, SP 05508270 Brazil; 4 Zoology Laboratory, Stabilization Unit, Laguna, UDESC, Av. Colombo Machado Salles 1873, Laguna, SC 88790-000 Brazil; 5 R3 Animal Association, Rod. João Gualberto Soares 11.000, Florianópolis, SC 88060-000 Brazil; 6 Marine Tetrapod Ecology and Conservation Laboratory, Marine Animal Stabilization Unit, São Francisco do Sul, University of the Joinville Region, UNIVILLE, Rod. Duque de Caxias 6.365, São Francisco do Sul, SC 89240-000 Brazil; 7 LIBGeo, UNIVALI, Rua Uruguai 458, Itajaí, SC 88301-902 Brazil; 8 Marine Animal Stabilization Unit, University of Vale do Itajaí - Univali BR, R Maria Emilia, 90, Armação, Penha, SC 88385-000 Brazil; 9 Australis Institute, Av. Atlantica, Sn, Praia de Itapirubá Norte, Imbituba, SC 88780-000 Brazil; 10 Albert Einstein Israelite Hospital, Av. Albert Einstein, 627/701, São Paulo, SP 05652-900 Brazil; 11 BSL-3 Laboratory of Virology and Applied Biotechnology, Department of Genetics, Evolution and Bioagents, Institute of Biology, University of Campinas – UNICAMP, P.O.Box: 6109, Campinas, SP 13083-862 Brazil; 12 BSL3+ Laboratory of Clinical and Molecular Virology, Institute of Biomedical Sciences, University of São Paulo, Av. Prof. Lineu Prestes 1374, São Paulo, SP 05508-000 Brazil; 13 Pasteur Institute of São Paulo/USP, University of São Paulo, São Paulo, 05508-020 Brazil; 14 LIM03/07, Institute of Tropical Medicine and Clinics Hospital, University of São Paulo School of Medicine, São Paulo, 05403-000 Brazil; 15 Wildlife Health Laboratory, College of Veterinary Medicine, Konkuk University, 120 Neungdong-Ro, Gwangjin-Gu, Seoul, 05029 Republic of Korea; 16 Konkuk University Zoonotic Diseases Research Center, College of Veterinary Medicine, Konkuk University, 120 Neungdong-Ro, Gwangjin-Gu, Seoul, 05029 Republic of Korea

**Keywords:** High pathogenicity avian influenza, Whole genome sequencing, Marine mammal, South America, Virus, Public health

## Abstract

Clade 2.3.4.4b highly pathogenic avian influenza (HPAI) H5N1 virus was detected in the South American sea lions found dead in Santa Catarina, Brazil, in October 2023. Whole genome sequencing and comparative phylogenetic analysis were conducted to investigate the origin, genetic diversity, and zoonotic potentials of the H5N1 viruses. The H5N1 viruses belonged to the genotype B3.2 of clade 2.3.4.4b H5N1 virus, which was identified in North America and disseminated to South America. They have acquired new amino acid substitutions related to mammalian host affinity. Our study provides insights into the genetic landscape of HPAI H5N1 viruses in Brazil, highlighting the continuous evolutionary processes contributing to their possible adaptation to mammalian hosts.

**Supplementary Information:**

The online version contains supplementary material available at 10.1186/s12917-024-04137-1.

## Introduction, methods, and results

The recent panzootic of the clade 2.3.4.4 b highly pathogenic avian influenza (HPAI) A (H5N1) virus caused massive outbreaks including wild birds, poultry, and mammalian species all around the globe since 2021 [1]. Particularly, mass mortality of coastal sea birds [2–4] and marine mammals caused by the HPAI H5N1 virus were reported from South America including Peru and Chile [4, 5]. In Brazil, the virus was identified for the first time in May 2023 in seabirds in the Southeast region and in the state of Santa Catarina in June 2023. Increase in detections of H5N1 virus in land and marine mammals are of wildlife and public health concern, as the viruses can rapidly evolve and adapt to mammals [3]. Here, we report the detection of clade 2.3.4.4 b HPAI H5N1 virus from South American sea lions (*Otaria flavescens*) in Santa Catarina, Brazil, during October of 2023. We conducted whole genome sequencing (WGS) and comparative phylogenetic analysis, to investigate the molecular epidemiology, genetic diversity, and mammalian adaptation markers of the H5N1 viruses.

**Fig. 1:**
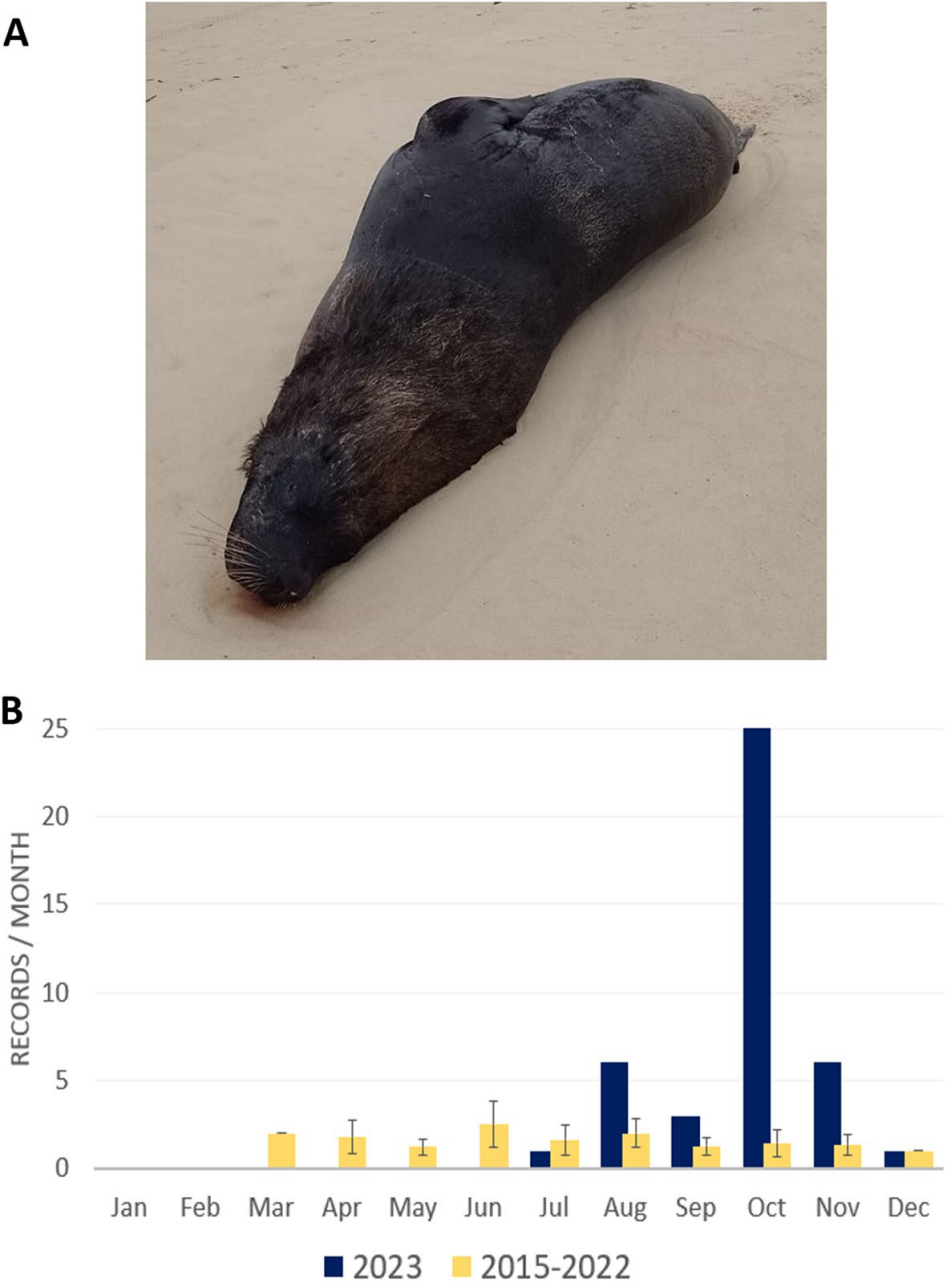
Atypical mortality of South American Sea Lion in Brazil. **A** South American Sea lion carcass in the beach of Florianópolis, **B** Sea lion mortality observed along the coastline of Santa Catarina State, Brazil throughout the year 2023 compared to the cumulative period of the previous seven years (2015–2022). Values for 2015–2022 are means and standard deviations for each month

A sudden surge of mortality in South American sea lions* (Otaria flavescens)* was recorded in October in the State of Santa Catarina, Brazil when compared to that of the previous years (Fig. 1). Most of the carcasses were at advanced decomposition without apparent gross lesion (SIMBA, 2023). To investigate if this unusual mortality was related to H5N1 virus, tissue samples of the digestive and respiratory tract were collected from October 10th to 15th, 2023. Additionally, to investigate the potential transmission among viruses infecting birds and marine mammals in the same geographic area, we collected cloacal swab samples from 190 seabirds (Fig. 1). Birds were found weakened on the beaches and taken for treatment in the Santa Catarina veterinary care network, stabilization, and rehabilitation centers. They were from five different orders and 17 species as follows: 101 Charadriiformes (*Larus dominicanus, Rynchops niger*, *Sterna hirundinacea*, *Sterna hirundo* and *Thalasseus acuflavidus*), 75 Sphenisciformes (*Spheniscus magellanicus*), 23 Suliformes (*Fregata magnificens*, *Phalacrocorax brasilianus* and *Sula leucogaster*), eight Procellariiformes (*Thalassarche melanophris*, *Pterodroma sp.*, *Puffinus puffinus* and *Procellaria aequinoctialis*), and two Pelecaniformes (*Phimosus infuscatus*). Samples were collected and stored in viral transport media by the Santos Basin Beach Monitoring Project, PMP-BS, under license ABIO 240/2015. This project has been continuously monitoring nearly the entire coastline of Santa Catarina state since August 2015, and the analyzed samples were from June 20th to December 4th, 2023. Total RNA was extracted using a MagMax core nucleic acid purification kit (ThermoFisher, California, USA) and screened to influenza A viruses by RT-qPCR targeting the matrix gene [6]. Data on the positive birds for HPAI H5N1 in the area was also collected from public data provided by the Brazilian Ministry of Agriculture (https://mapa-indicadores.agricultura.gov.br/publico/extensions/SRN/SRN.html).

For each positive sample, amplification of all 8 target genes was conducted using the *SuperScript* IV *Reverse Transcriptase kit* (Thermo Fisher Scientific, USA) with the universal primers as previously described [7]. For next generation sequencing (NGS), library preparation was done using the Nextera XT DNA kit (Illumina) and run on NextSeq 550 (Illumina, San Diego, California, USA) with NextSeq 500/550 Mid Output Kit v2 (300-cycles). Genome assembly was performed and visualized using the map to reference tool in Geneious Prime software (https://www.geneious.com). Consensus avian influenza virus (AIV) genome sequences identified in the current study have been deposited in GenBank with accession numbers PP094668 to PP094683.

**Fig. 2:**
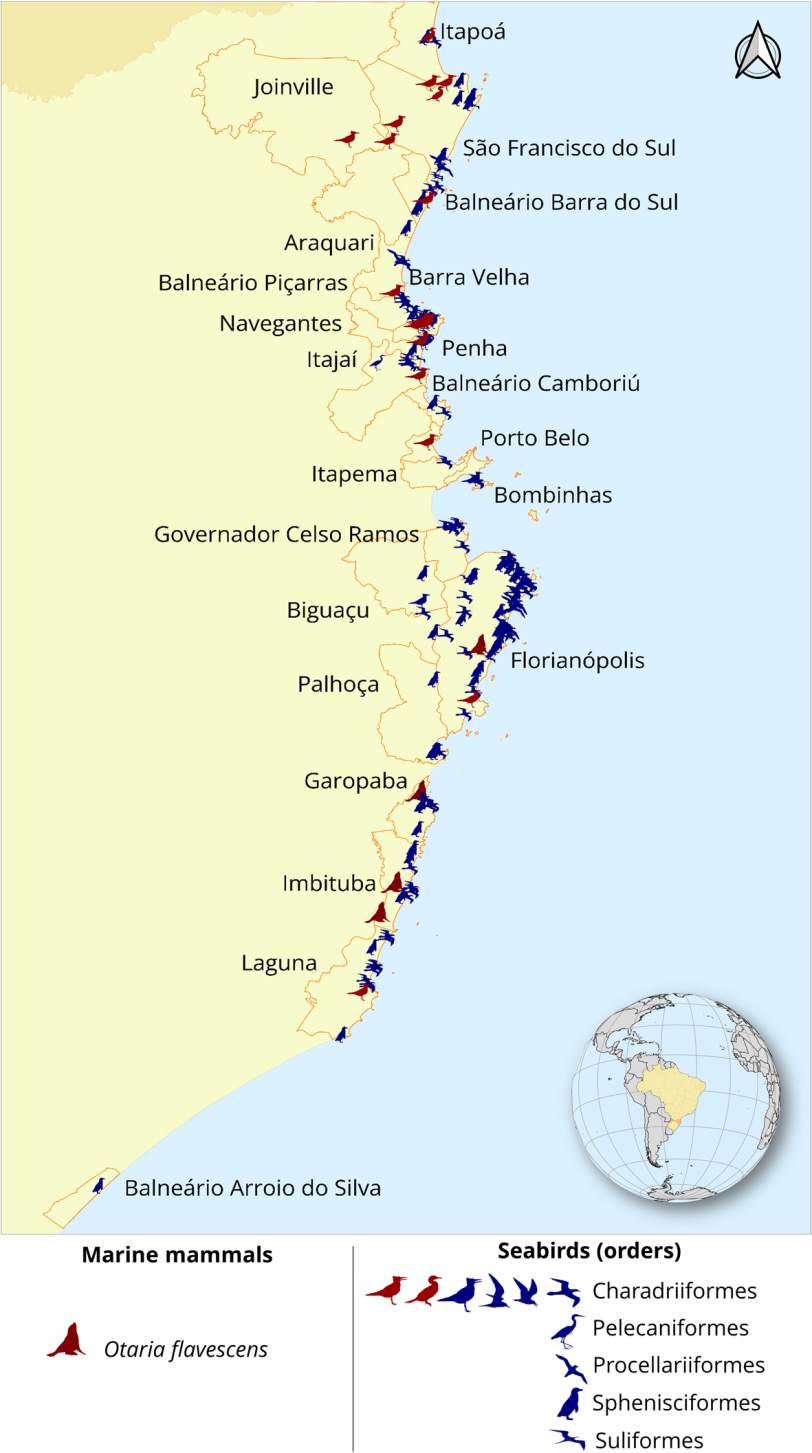
Map of all tested animals (seabirds and sea lions) in Santa Catarina, Brazil, from June 20th to December 4th, 2023. Red symbols indicate positive results for HPAI

The complete coding sequences of the viruses were submitted to the GISAID BLAST database (https://gisaid.org/) for the query of top 250 BLAST results of all eight genes. Result sequences were downloaded and aligned using the MAFFT versions 7.520 (https://mafft.cbrc.jp/alignment/software/) and sequences with 100% of nucleotide identity were eliminated using the ElimDupes (https://www.hiv.lanl.gov/content/sequence/elimdupesv2/elimdupes.html). Maximum-likelihood (ML) trees were constructed using the RaxML v8.0 software using rapid bootstrapping replicate of 1000 trees to search for best-scoring ML tree with general time-reversible + Gamma nucleotide substitution model. ML trees were visualized using Interactive Tree of Life v6 (https://itol.embl.de/, accessed on 11 December 2023) [8]. Genotype of the viruses were determined by examining ML phylogeny topology and query result using the GenoFlu (https://github.com/USDA-VS/GenoFLU), which classifies segments according to Youk et al. [9] and was originally designed to describe H5NX 2.3.4.4b genotypes found in the Americas. Molecular markers associated with mammalian adaptation were screened using the FluSurver mutations app (http://flusurver.bii.a-star.edu.sg) and previous publications [10]. For the estimation of the time to the most recent ancestor (MRCA) harboring the mutation, genetic reassortment was excluded and concatenated complete genome sequences of South American H5N1 viruses of were used in combination with Bayesian stochastic search variable selection using BEAST package 1.10.4 as previously described [11, 12]. For all phylogeographic analyses an uncorrelated log-normal distribution relaxed-clock method with a Hasegawa, Kishino, and Yano nucleotide substitution model and Bayesian skyride coalescent prior was used. Five out of eight samples from the respiratory tract of four sea lions were tested positive by the RT-qPCR test using the AIV matrix gene as the target. Among the positive samples, we sequenced two samples that had a cycle threshold (Ct) value ranging below 25. We obtained complete coding genome sequences of two viruses and named as A/South American sea lion/Brazil/OF-358R/2023(H5N1) and A/South American sea lion/Brazil/OF-359R/2023(H5N1) (hereafter, OF-358R/23 and OF-359R/23). Only one sample from the digestive tract (animal OF-359) was positive by the RT- qPCR with a Ct value of 33. None of the birds collected by our group tested positive for the RT- qPCR specific for AIV. Ten outbreaks in birds and one in sea lions were reported in Santa Catarina from September to October by the Official Veterinary Service (OVS) of the Ministry of Agriculture (Fig. 2). Maximum-likelihood (ML) phylogenies constructed using the internal genes revealed that the OF-358R/23 and OF-359R/23 viruses clustered with the previously reported South American clade 2.3.4.4 b H5N1 HPAIVs (Supplementary Figure S1). Genotype BLAST search using the GenoFlu also identified the eight genes as genotype B3.2 with less than 1% nucleotide difference [9]. No evidence of genetic reassortment was observed in these viruses [3]. Hemagglutinin (HA) cleavage site of the viruses possessed a HPAI motif, PLREKRRKR/GLF, which is identical to the recent clade 2.3.4.4 b HPAI H5N1 viruses found in South America.

Amino acid substitutions related to mammalian adaptation were observed in the genomes of OF-358R/23 and OF-359R/23 viruses. Notably, amino acid substitutions of Q591K and D701N in PB2 gene, R57Q in PA gene, and V226T in NS were newly acquired when compared to the HPAI H5N1 isolates previously reported from wild birds in Brazil (Table 1) [13, 14]. These amino acid substitutions found in the H5N1 viruses are known to increase virulence, transmissibility, or replication in mammalian host species.

**Table 1:**
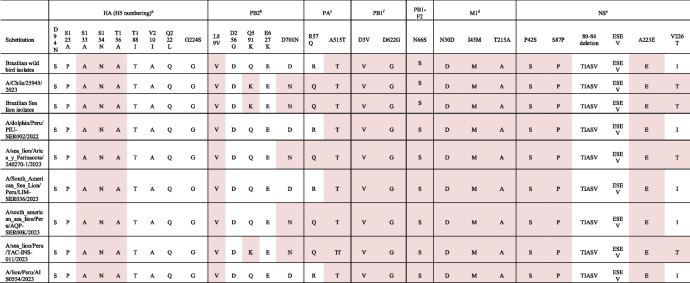
Amino acid substitutions of clade 2.3.4.4b H5N1 HPAIV. Molecular markers associated with increased virulence in mammals were highlighted in pink

^a^D94N, S123P, S133A, S154N, T156A, T188I, Q222L, and G224S mutations in HA are known to be associated with increased binding to human-like receptor (α-2,6 sialic acid)

^b^L89V and D256G mutations in PB2 are known to be associated with increased virulence in mice. Q591K, E627K, and D701N mutations in PB2 are known to be associated with increased viral replication in mammals

^c^R57Q mutation in PA is known to be associated with host specificity shift. A515T mutation in PA is known to be associated with H5 transmissibility in ferrets

^d^N30D, I43M, T215A mutations in M1 are known to be associated with increased virulence in mice

^e^P42S, 80–84 deletion, A223E, V226T, and ESEV PDZ binding motif mutations in NS are known to be associated with increased virulence in mice. S87P is known to be associated with host specificity shift

^f^PB1: D3V Increased polymerase activity and viral replication in avian and mammalian cell lines, D622G Increased polymerase activity and virulence in mice and in PB1-F2: N66S Enhanced replication, virulence and antiviral response in mice

**Fig. 3:**
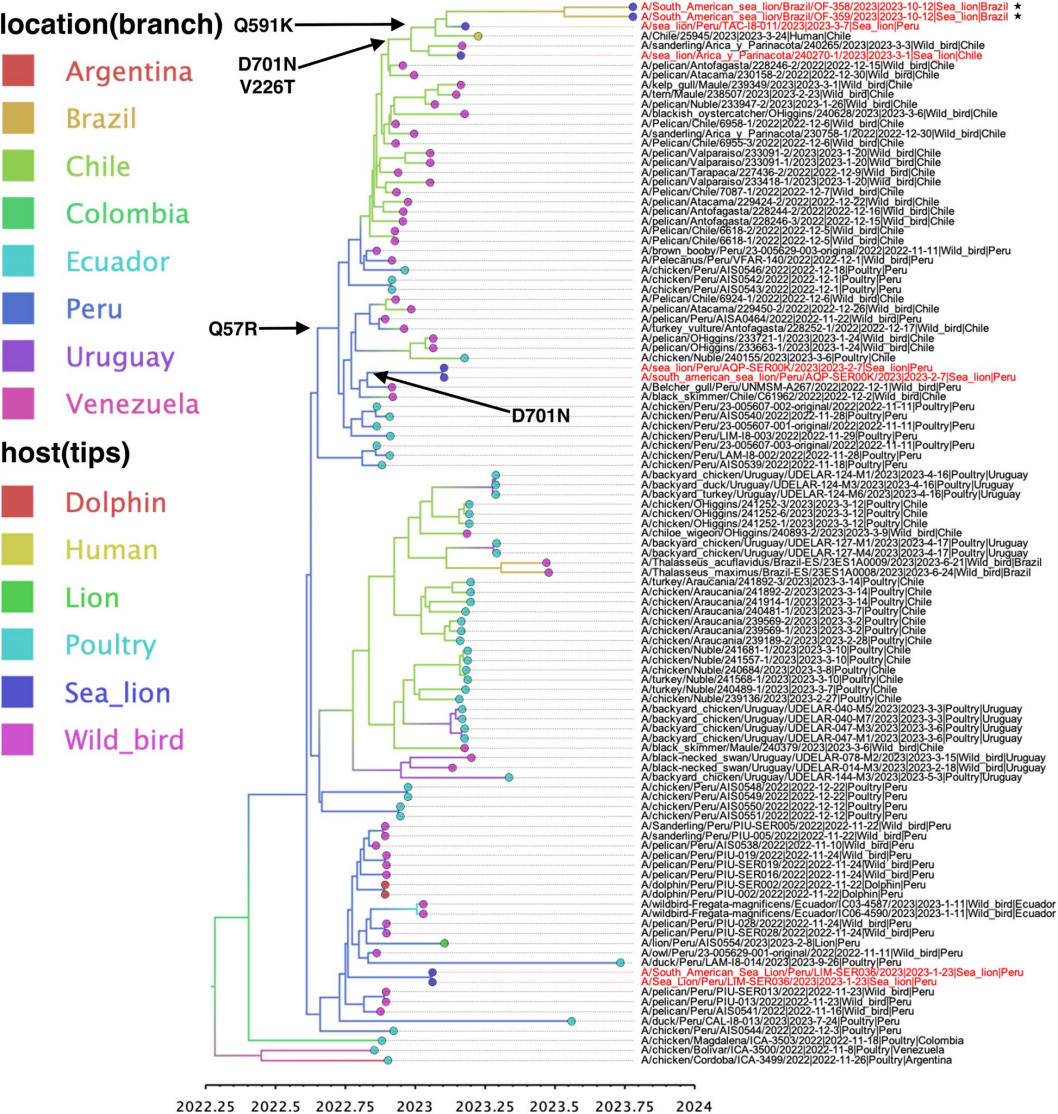
Time-scaled maximum clade credibility phylogenetic tree of concatenated eight gene segments of avian influenza viruses. Node bars in blue represent 95% Bayesian credible intervals (posterior probability > 0.5). The horizontal axis defines the time scale in decimal years. The taxa of H5N1 HPAI from sea lions in Brazil are indicated in red. The ancestral node of suspected emergence of the amino acid substitutions are labelled using black arrow

OF-358R/23 and OF-359R/23 viruses grouped in the same main cluster grouping the human isolate originating from Chile and sea lion isolates originating from Peru and Chile (Fig. 3 and Supplementary Fig. 1 and 2). For better resolution of the estimation of MRCA of the unique amino acid substitutions, concatenated genome sequences of all eight influenza genes were used. R57Q in PA gene first arose around September 23rd, 2022 (95% highest posterior density, HPD: September 5th, 2022 to October 11th, 2022). D701N in PB2 gene most likely occurred twice in a separate instance, the first occurrence was around December 26th, 2022 (95% HPD: December 8th, 2022 to January 13th, 2023), the second occurrence was also in sea lion isolate from Peru around February 4th, 2023 (95% HPD: January 30th, 2023 to February 7th, 2023) (Fig. 3). V226T in NS gene was first observed during the same period as the first appearance of D701N in PB2. Q591K in PB2 emerged around January 27th, 2023 (95% HPD: January 9th, 2023 to February 13th, 2023) (Fig. 3).

## Discussion

Here, we sequenced and analyzed two complete genome sequences of clade 2.3.4.4 b HPAI H5N1 viruses detected from carcasses of South American sea lions. Phylogenetic analysis of the whole genome sequences indicated no evidence of reassortment from the genotype B3.2 initially introduced to South America. Most probable transmission route of the H5N1 virus is from North America to South America by wild birds, subsequently disseminated to marine mammals. Previous studies have suggested the possibility of establishment and transmission among marine mammals along South America American coastlines [3, 5]. Based on the obtained data, it is unclear if the OF-358R/23 and OF-359R/23 viruses were originated from marine mammals or wild birds since there was no direct ancestral genome sequences available in public databases.

OF-358R/23 and OF-359R/23 showed close genetic relationship with the human isolate from Chile and Sea lion isolate from Peru, which also harbored amino acid substitutions such as Q591K, D701N, and V226T. Q57R emerged in the late 2022, at least a year before the detection of OF-358R/23 and OF-359R/23 (Fig. 3). As previously described, our analysis supported that D701N have emerged in two separate instances of sea lion virus clusters [3]. Convergent mutations like D701N highlight the need for the close monitoring of mutations arising in the viruses. Bayesian inference using the concatenated complete genome sequences revealed that the mutations in OF-358R/23 and OF-359R/23 were sustained after their emergence and has been circulating since the late 2022 and early 2023. The accumulation of these mutations may have aided in the adaptation of the H5N1 viruses to possible be transmitted among sea lion population along the coast of South America and increase their potential for zoonotic transmission.

Comparative genome analysis with the recent mammalian origin viruses from South America revealed that the amino acid substitutions of Q591K and D704N in PB2, R57Q in PA, and V226T in NS were not universally found in all South American mammalian viruses, indicating that mutations associated with mammalian affinity may be accumulating. Increased detection of HPAI in marine mammals and the presence of such mammalian molecular markers raise concern as increased affinity to mammalian host species pose as a significant threat to both mammalian wildlife and public health. Enhanced surveillance and continued monitoring effort is needed to elucidate the evidence of transmission among marine mammals and be wary of the potential spillover into human population.

### Supplementary Information

Supplementary Material 1: Supplementary Figure 1. Maximum-likelihood tree constructed using RAxML v8.0 using the complete coding nucleotide sequences of (A) polymerase basic protein 2, (B) polymerase basic protein 1, (C) polymerase acidic protein, (D) hemagglutinin protein, (E) nucleoprotein, (F) neuraminidase protein, (G) matrix protein, and (H) non-structural protein. Blue taxa label indicates all seal lion origin virus. Yellow shade highlights Brazilian viruses. Numerical values at the nodes represent 1,000 bootstrap replicate value (%). Bootstrap value < 70 was removed from the tree.

Supplementary Material 2: Supplementary Figure 2. Phylogeography of South American H5N1 HPAI using the concatenated sequences of all influenza genome segment. The horizontal axis defines the time scale in decimal years. Branch colors represent different countries as shown in figure legend (AR: Argentina, BO: Bolivar, BR: Brazil, CH: Chile, CO: Columbia, EC: Ecuador, PR: Peru, and UR: Uruguay).

Supplementary Material 3: Supplementary Table 1. Summary of tested animals on the coast of Santa Catarina State.

## Data Availability

The dataset presented in this study can be found in online repositories. The names of the repository/repositories and accession number(s) can be found in GeneBank Database under the accession numbers PP094668 to PP094683.
