# Supplementary material for: Mortality in sea lions is associated with the introduction of the H5N1 clade 2.3.4.4b virus in Brazil October 2023: whole genome sequencing and phylogenetic analysis
Source: BMC Vet Res. 2024 Jul 2;20:285. doi: 10.1186/s12917-024-04137-1 (PMC11221036; doi:10.1186/s12917-024-04137-1)
Supplement: Supplementary file 1 — Supplementary Material 1: Supplementary Figure 1. Maximum-likelihood tree constructed using RAxML v8.0 using the complete coding nucleotide sequences of (A) polymerase basic protein 2, (B) polymerase basic protein 1, (C) polymerase acidic protein, (D) hemagglutinin protein, (E) nucleoprotein, (F) neuraminidase protein, (G) matrix protein, and (H) non-structural protein. Blue taxa label indicates all seal lion origin virus. Yellow shade highlights Brazilian viruses. Numerical values at the nodes represent 1,000 bootstrap replicate value (%). Bootstrap value < 70 was removed from the tree. [file 12917_2024_4137_MOESM1_ESM.pdf]

Supplementary Material

Supplementary Material was uploaded separately on submission.

Journal: BMC Veterinary Research

Title: Mortality in Sea Lions is associated with the introduction of the H5N1 clade 2.3.4.4b virus in Brazil, October 2023: Whole genome sequencing and phylogenetic analysis

Authors: Andreina Carvalho de Araújo, Andrew Yong Cho, Laura Morais Nascimento Silva, Thais Camilo Corrêa, Gabriela Cristini de Souza, Adriana Silva Albuquerque, Eduardo Macagnan, Cristiane K. M. Kolesnikvoas, Rafael Meurer, Jenyffer Vierheller Vieira, Giulia Gaglianone Lemos, André Silva Barreto, Jeferson Luis Dick, Karina Rejane Groch, Pedro Volkmer de Castilho, Deyvid Amgarten, Fernanda Malta, Michael Miller, Erick G. Dorlass, Soledad Palameta, Sun-Hak Lee, Clarice Weis Arns, Edison L. Durigon, João Renato R. Pinho, Dong-Hun Lee, Helena Lage Ferreira

Supplementary Figure 1. Maximum-likelihood tree constructed using RAxML v8.0 using the complete coding nucleotide sequences of (A) polymerase basic protein 2, (B) polymerase basic protein 1, (C) polymerase acidic protein, (D) hemagglutinin protein, (E) nucleoprotein, (F) neuraminidase protein, (G) matrix protein, and (H) non-structural protein. Blue taxa label indicates all seal lion origin virus. Yellow shade highlights Brazilian viruses. Numerical values at the nodes represent 1,000 bootstrap replicate value (%). Bootstrap value < 70 was removed from the tree.

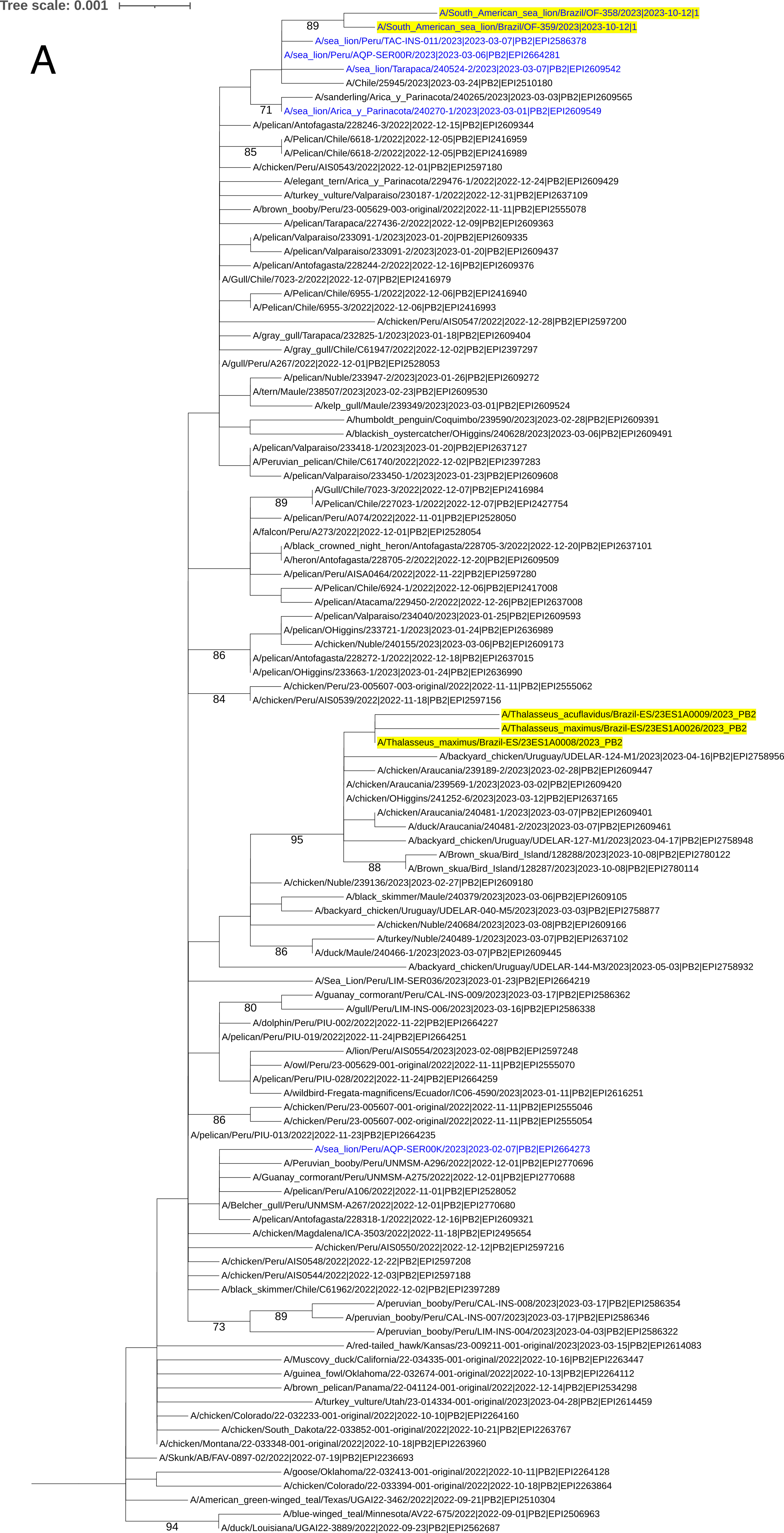
Tree scale: 0.001
A
89
A/South_American_sea_lion/Brazil/OF-358/2023|2023-10-12|1
A/South_American_sea_lion/Brazil/OF-359/2023|2023-10-12|1
A/sea_lion/Peru/TAC-INS-011/2023|2023-03-07|PB2|EPI2586378
A/sea_lion/Peru/AQP-SER00R/2023|2023-03-06|PB2|EPI2664281
A/sea_lion/Tarapaca/240524-2/2023|2023-03-07|PB2|EPI2609542
A/Chile/25945/2023|2023-03-24|PB2|EPI2510180
A/sanderling/Arica_y_Parinacota/240265/2023|2023-03-03|PB2|EPI2609565
71
A/sea_lion/Arica_y_Parinacota/240270-1/2023|2023-03-01|PB2|EPI2609549
A/pelican/Antofagasta/228246-3/2022|2022-12-15|PB2|EPI2609344
A/Pelican/Chile/6618-1/2022|2022-12-05|PB2|EPI2416959
85
A/Pelican/Chile/6618-2/2022|2022-12-05|PB2|EPI2416989
A/chicken/Peru/AIS0543/2022|2022-12-01|PB2|EPI2597180
A/elegant_tern/Arica_y_Parinacota/229476-1/2022|2022-12-24|PB2|EPI2609429
A/turkey_vulture/Valparaiso/230187-1/2022|2022-12-31|PB2|EPI2637109
A/brown_booby/Peru/23-005629-003-original/2022|2022-11-11|PB2|EPI2555078
A/pelican/Tarapaca/227436-2/2022|2022-12-09|PB2|EPI2609363
A/pelican/Valparaiso/233091-1/2023|2023-01-20|PB2|EPI2609335
A/pelican/Valparaiso/233091-2/2023|2023-01-20|PB2|EPI2609437
A/pelican/Antofagasta/228244-2/2022|2022-12-16|PB2|EPI2609376
A/Gull/Chile/7023-2/2022|2022-12-07|PB2|EPI2416979
A/Pelican/Chile/6955-1/2022|2022-12-06|PB2|EPI2416940
A/Pelican/Chile/6955-3/2022|2022-12-06|PB2|EPI2416993
A/chicken/Peru/AIS0547/2022|2022-12-28|PB2|EPI2597200
A/gray_gull/Tarapaca/232825-1/2023|2023-01-18|PB2|EPI2609404
A/gray_gull/Chile/C61947/2022|2022-12-02|PB2|EPI2397297
A/gull/Peru/A267/2022|2022-12-01|PB2|EPI2528053
A/pelican/Nuble/233947-2/2023|2023-01-26|PB2|EPI2609272
A/tern/Maule/238507/2023|2023-02-23|PB2|EPI2609530
A/kelp_gull/Maule/239349/2023|2023-03-01|PB2|EPI2609524
A/humboldt_penguin/Coquimbo/239590/2023|2023-02-28|PB2|EPI2609391
A/blackish_oystercatcher/OHiggins/240628/2023|2023-03-06|PB2|EPI2609491
A/pelican/Valparaiso/233418-1/2023|2023-01-20|PB2|EPI2637127
A/Peruvian_pelican/Chile/C61740/2022|2022-12-02|PB2|EPI2397283
A/pelican/Valparaiso/233450-1/2023|2023-01-23|PB2|EPI2609608
A/Gull/Chile/7023-3/2022|2022-12-07|PB2|EPI2416984
89
A/Pelican/Chile/227023-1/2022|2022-12-07|PB2|EPI2427754
A/pelican/Peru/A074/2022|2022-11-01|PB2|EPI2528050
A/falcon/Peru/A273/2022|2022-12-01|PB2|EPI2528054
A/black_crowned_night_heron/Antofagasta/228705-3/2022|2022-12-20|PB2|EPI2637101
A/heron/Antofagasta/228705-2/2022|2022-12-20|PB2|EPI2609509
A/pelican/Peru/AISA0464/2022|2022-11-22|PB2|EPI2597280
A/Pelican/Chile/6924-1/2022|2022-12-06|PB2|EPI2417008
A/pelican/Atacama/229450-2/2022|2022-12-26|PB2|EPI2637008
A/pelican/Valparaiso/234040/2023|2023-01-25|PB2|EPI2609593
A/pelican/OHiggins/233721-1/2023|2023-01-24|PB2|EPI2636989
A/chicken/Nuble/240155/2023|2023-03-06|PB2|EPI2609173
86
A/pelican/Antofagasta/228272-1/2022|2022-12-18|PB2|EPI2637015
A/pelican/OHiggins/233663-1/2023|2023-01-24|PB2|EPI2636990
A/chicken/Peru/23-005607-003-original/2022|2022-11-11|PB2|EPI2555062
84
A/chicken/Peru/AIS0539/2022|2022-11-18|PB2|EPI2597156
A/Thalasseus_acuflavidus/Brazil-ES/23ES1A0009/2023_PB2
A/Thalasseus_maximus/Brazil-ES/23ES1A0026/2023_PB2
A/Thalasseus_maximus/Brazil-ES/23ES1A0008/2023_PB2
A/backyard_chicken/Uruguay/UDELAR-124-M1/2023|2023-04-16|PB2|EPI2758956
A/chicken/Araucania/239189-2/2023|2023-02-28|PB2|EPI2609447
A/chicken/Araucania/239569-1/2023|2023-03-02|PB2|EPI2609420
A/chicken/OHiggins/241252-6/2023|2023-03-12|PB2|EPI2637165
A/chicken/Araucania/240481-1/2023|2023-03-07|PB2|EPI2609401
A/duck/Araucania/240481-2/2023|2023-03-07|PB2|EPI2609461
95
A/backyard_chicken/Uruguay/UDELAR-127-M1/2023|2023-04-17|PB2|EPI2758948
A/Brown_skua/Bird_Island/128288/2023|2023-10-08|PB2|EPI2780122
88
A/Brown_skua/Bird_Island/128287/2023|2023-10-08|PB2|EPI2780114
A/chicken/Nuble/239136/2023|2023-02-27|PB2|EPI2609180
A/black_skimmer/Maule/240379/2023|2023-03-06|PB2|EPI2609105
A/backyard_chicken/Uruguay/UDELAR-040-M5/2023|2023-03-03|PB2|EPI2758877
A/chicken/Nuble/240684/2023|2023-03-08|PB2|EPI2609166
A/turkey/Nuble/240489-1/2023|2023-03-07|PB2|EPI2637102
86
A/duck/Maule/240466-1/2023|2023-03-07|PB2|EPI2609445
A/backyard_chicken/Uruguay/UDELAR-144-M3/2023|2023-05-03|PB2|EPI2758932
A/Sea_Lion/Peru/LIM-SER036/2023|2023-01-23|PB2|EPI2664219
A/guanay_cormorant/Peru/CAL-INS-009/2023|2023-03-17|PB2|EPI2586362
80
A/gull/Peru/LIM-INS-006/2023|2023-03-16|PB2|EPI2586338
A/dolphin/Peru/PIU-002/2022|2022-11-22|PB2|EPI2664227
A/pelican/Peru/PIU-019/2022|2022-11-24|PB2|EPI2664251
A/lion/Peru/AIS0554/2023|2023-02-08|PB2|EPI2597248
A/owl/Peru/23-005629-001-original/2022|2022-11-11|PB2|EPI2555070
A/pelican/Peru/PIU-028/2022|2022-11-24|PB2|EPI2664259
A/wildbird-Fregata-magnificens/Ecuador/IC06-4590/2023|2023-01-11|PB2|EPI2616251
A/chicken/Peru/23-005607-001-original/2022|2022-11-11|PB2|EPI2555046
86
A/chicken/Peru/23-005607-002-original/2022|2022-11-11|PB2|EPI2555054
A/pelican/Peru/PIU-013/2022|2022-11-23|PB2|EPI2664235
A/sea_lion/Peru/AQP-SER00K/2023|2023-02-07|PB2|EPI2664273
A/Peruvian_booby/Peru/UNMSM-A296/2022|2022-12-01|PB2|EPI2770696
A/Guanay_cormorant/Peru/UNMSM-A275/2022|2022-12-01|PB2|EPI2770688
A/pelican/Peru/A106/2022|2022-11-01|PB2|EPI2528052
A/Belcher_gull/Peru/UNMSM-A267/2022|2022-12-01|PB2|EPI2770680
A/pelican/Antofagasta/228318-1/2022|2022-12-16|PB2|EPI2609321
A/chicken/Magdalena/ICA-3503/2022|2022-11-18|PB2|EPI2495654
A/chicken/Peru/AIS0550/2022|2022-12-12|PB2|EPI2597216
A/chicken/Peru/AIS0548/2022|2022-12-22|PB2|EPI2597208
A/chicken/Peru/AIS0544/2022|2022-12-03|PB2|EPI2597188
A/black_skimmer/Chile/C61962/2022|2022-12-02|PB2|EPI2397289
A/peruvian_booby/Peru/CAL-INS-008/2023|2023-03-17|PB2|EPI2586354
89
A/peruvian_booby/Peru/CAL-INS-007/2023|2023-03-17|PB2|EPI2586346
73
A/peruvian_booby/Peru/LIM-INS-004/2023|2023-04-03|PB2|EPI2586322
A/red-tailed_hawk/Kansas/23-009211-001-original/2023|2023-03-15|PB2|EPI2614083
A/Muscovy_duck/California/22-034335-001-original/2022|2022-10-16|PB2|EPI2263447
A/guinea_fowl/Oklahoma/22-032674-001-original/2022|2022-10-13|PB2|EPI2264112
A/brown_pelican/Panama/22-041124-001-original/2022|2022-12-14|PB2|EPI2534298
A/turkey_vulture/Utah/23-014334-001-original/2023|2023-04-28|PB2|EPI2614459
A/chicken/Colorado/22-032233-001-original/2022|2022-10-10|PB2|EPI2264160
A/chicken/South_Dakota/22-033852-001-original/2022|2022-10-21|PB2|EPI2263767
A/chicken/Montana/22-033348-001-original/2022|2022-10-18|PB2|EPI2263960
A/Skunk/AB/FAV-0897-02/2022|2022-07-19|PB2|EPI2236693
A/goose/Oklahoma/22-032413-001-original/2022|2022-10-11|PB2|EPI2264128
A/chicken/Colorado/22-033394-001-original/2022|2022-10-18|PB2|EPI2263864
A/American_green-winged_teal/Texas/UGAI22-3462/2022|2022-09-21|PB2|EPI2510304
A/blue-winged_teal/Minnesota/AV22-675/2022|2022-09-01|PB2|EPI2506963
94
A/duck/Louisiana/UGAI22-3889/2022|2022-09-23|PB2|EPI2562687

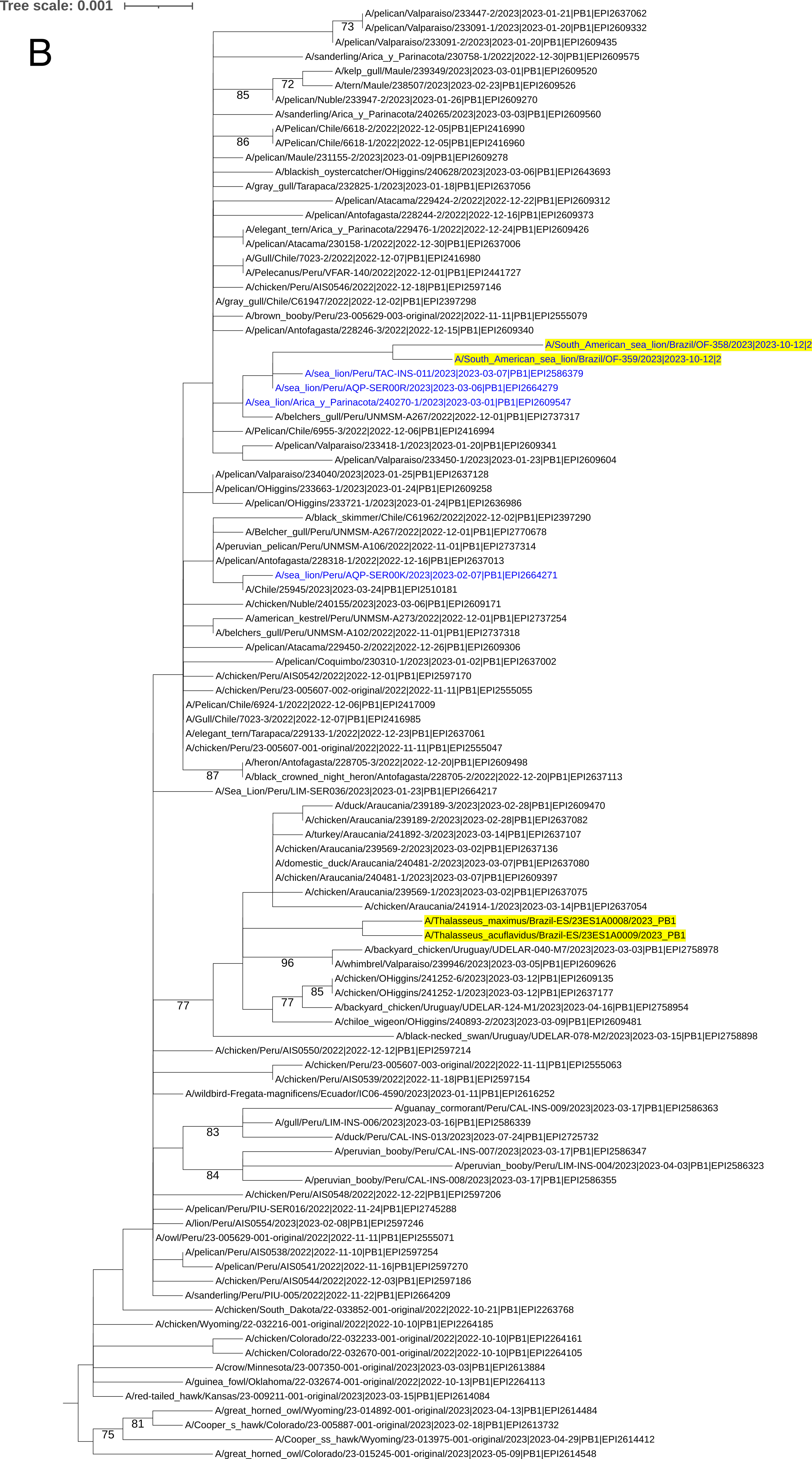
Tree scale: 0.001
B
A/pelican/Valparaiso/233447-2/2023|2023-01-21|PB1|EPI2637062
73
A/pelican/Valparaiso/233091-1/2023|2023-01-20|PB1|EPI2609332
A/pelican/Valparaiso/233091-2/2023|2023-01-20|PB1|EPI2609435
A/sanderling/Arica_y_Parinacota/230758-1/2022|2022-12-30|PB1|EPI2609575
A/kelp_gull/Maule/239349/2023|2023-03-01|PB1|EPI2609520
72
A/tern/Maule/238507/2023|2023-02-23|PB1|EPI2609526
85
A/pelican/Nuble/233947-2/2023|2023-01-26|PB1|EPI2609270
A/sanderling/Arica_y_Parinacota/240265/2023|2023-03-03|PB1|EPI2609560
A/Pelican/Chile/6618-2/2022|2022-12-05|PB1|EPI2416990
86
A/Pelican/Chile/6618-1/2022|2022-12-05|PB1|EPI2416960
A/pelican/Maule/231155-2/2023|2023-01-09|PB1|EPI2609278
A/blackish_oystercatcher/OHiggins/240628/2023|2023-03-06|PB1|EPI2643693
A/gray_gull/Tarapaca/232825-1/2023|2023-01-18|PB1|EPI2637056
A/pelican/Atacama/229424-2/2022|2022-12-22|PB1|EPI2609312
A/pelican/Antofagasta/228244-2/2022|2022-12-16|PB1|EPI2609373
A/elegant_tern/Arica_y_Parinacota/229476-1/2022|2022-12-24|PB1|EPI2609426
A/pelican/Atacama/230158-1/2022|2022-12-30|PB1|EPI2637006
A/Gull/Chile/7023-2/2022|2022-12-07|PB1|EPI2416980
A/Pelecanus/Peru/VFAR-140/2022|2022-12-01|PB1|EPI2441727
A/chicken/Peru/AIS0546/2022|2022-12-18|PB1|EPI2597146
A/gray_gull/Chile/C61947/2022|2022-12-02|PB1|EPI2397298
A/brown_booby/Peru/23-005629-003-original/2022|2022-11-11|PB1|EPI2555079
A/pelican/Antofagasta/228246-3/2022|2022-12-15|PB1|EPI2609340
A/South_American_sea_lion/Brazil/OF-358/2023|2023-10-12|2
A/South_American_sea_lion/Brazil/OF-359/2023|2023-10-12|2
A/sea_lion/Peru/TAC-INS-011/2023|2023-03-07|PB1|EPI2586379
A/sea_lion/Peru/AQP-SER00R/2023|2023-03-06|PB1|EPI2664279
A/sea_lion/Arica_y_Parinacota/240270-1/2023|2023-03-01|PB1|EPI2609547
A/belchers_gull/Peru/UNMSM-A267/2022|2022-12-01|PB1|EPI2737317
A/Pelican/Chile/6955-3/2022|2022-12-06|PB1|EPI2416994
A/pelican/Valparaiso/233418-1/2023|2023-01-20|PB1|EPI2609341
A/pelican/Valparaiso/233450-1/2023|2023-01-23|PB1|EPI2609604
A/pelican/Valparaiso/234040/2023|2023-01-25|PB1|EPI2637128
A/pelican/OHiggins/233663-1/2023|2023-01-24|PB1|EPI2609258
A/pelican/OHiggins/233721-1/2023|2023-01-24|PB1|EPI2636986
A/black_skimmer/Chile/C61962/2022|2022-12-02|PB1|EPI2397290
A/Belcher_gull/Peru/UNMSM-A267/2022|2022-12-01|PB1|EPI2770678
A/peruvian_pelican/Peru/UNMSM-A106/2022|2022-11-01|PB1|EPI2737314
A/pelican/Antofagasta/228318-1/2022|2022-12-16|PB1|EPI2637013
A/sea_lion/Peru/AQP-SER00K/2023|2023-02-07|PB1|EPI2664271
A/Chile/25945/2023|2023-03-24|PB1|EPI2510181
A/chicken/Nuble/240155/2023|2023-03-06|PB1|EPI2609171
A/american_kestrel/Peru/UNMSM-A273/2022|2022-12-01|PB1|EPI2737254
A/belchers_gull/Peru/UNMSM-A102/2022|2022-11-01|PB1|EPI2737318
A/pelican/Atacama/229450-2/2022|2022-12-26|PB1|EPI2609306
A/pelican/Coquimbo/230310-1/2023|2023-01-02|PB1|EPI2637002
A/chicken/Peru/AIS0542/2022|2022-12-01|PB1|EPI2597170
A/chicken/Peru/23-005607-002-original/2022|2022-11-11|PB1|EPI2555055
A/Pelican/Chile/6924-1/2022|2022-12-06|PB1|EPI2417009
A/Gull/Chile/7023-3/2022|2022-12-07|PB1|EPI2416985
A/elegant_tern/Tarapaca/229133-1/2022|2022-12-23|PB1|EPI2637061
A/chicken/Peru/23-005607-001-original/2022|2022-11-11|PB1|EPI2555047
A/heron/Antofagasta/228705-3/2022|2022-12-20|PB1|EPI2609498
87
A/black_crowned_night_heron/Antofagasta/228705-2/2022|2022-12-20|PB1|EPI2637113
A/Sea_Lion/Peru/LIM-SER036/2023|2023-01-23|PB1|EPI2664217
A/duck/Araucania/239189-3/2023|2023-02-28|PB1|EPI2609470
A/chicken/Araucania/239189-2/2023|2023-02-28|PB1|EPI2637082
A/turkey/Araucania/241892-3/2023|2023-03-14|PB1|EPI2637107
A/chicken/Araucania/239569-2/2023|2023-03-02|PB1|EPI2637136
A/domestic_duck/Araucania/240481-2/2023|2023-03-07|PB1|EPI2637080
A/chicken/Araucania/240481-1/2023|2023-03-07|PB1|EPI2609397
A/chicken/Araucania/239569-1/2023|2023-03-02|PB1|EPI2637075
A/chicken/Araucania/241914-1/2023|2023-03-14|PB1|EPI2637054
A/Thalasseus_maximus/Brazil-ES/23ES1A0008/2023_PB1
A/Thalasseus_acuflavidus/Brazil-ES/23ES1A0009/2023_PB1
A/backyard_chicken/Uruguay/UDELAR-040-M7/2023|2023-03-03|PB1|EPI2758978
96
A/whimbrel/Valparaiso/239946/2023|2023-03-05|PB1|EPI2609626
A/chicken/OHiggins/241252-6/2023|2023-03-12|PB1|EPI2609135
85
A/chicken/OHiggins/241252-1/2023|2023-03-12|PB1|EPI2637177
77
77
A/backyard_chicken/Uruguay/UDELAR-124-M1/2023|2023-04-16|PB1|EPI2758954
A/chiloe_wigeon/OHiggins/240893-2/2023|2023-03-09|PB1|EPI2609481
A/black-necked_swan/Uruguay/UDELAR-078-M2/2023|2023-03-15|PB1|EPI2758898
A/chicken/Peru/AIS0550/2022|2022-12-12|PB1|EPI2597214
A/chicken/Peru/23-005607-003-original/2022|2022-11-11|PB1|EPI2555063
A/chicken/Peru/AIS0539/2022|2022-11-18|PB1|EPI2597154
A/wildbird-Fregata-magnificens/Ecuador/IC06-4590/2023|2023-01-11|PB1|EPI2616252
A/guanay_cormorant/Peru/CAL-INS-009/2023|2023-03-17|PB1|EPI2586363
A/gull/Peru/LIM-INS-006/2023|2023-03-16|PB1|EPI2586339
83
A/duck/Peru/CAL-INS-013/2023|2023-07-24|PB1|EPI2725732
A/peruvian_booby/Peru/CAL-INS-007/2023|2023-03-17|PB1|EPI2586347
A/peruvian_booby/Peru/LIM-INS-004/2023|2023-04-03|PB1|EPI2586323
84
A/peruvian_booby/Peru/CAL-INS-008/2023|2023-03-17|PB1|EPI2586355
A/chicken/Peru/AIS0548/2022|2022-12-22|PB1|EPI2597206
A/pelican/Peru/PIU-SER016/2022|2022-11-24|PB1|EPI2745288
A/lion/Peru/AIS0554/2023|2023-02-08|PB1|EPI2597246
A/owl/Peru/23-005629-001-original/2022|2022-11-11|PB1|EPI2555071
A/pelican/Peru/AIS0538/2022|2022-11-10|PB1|EPI2597254
A/pelican/Peru/AIS0541/2022|2022-11-16|PB1|EPI2597270
A/chicken/Peru/AIS0544/2022|2022-12-03|PB1|EPI2597186
A/sanderling/Peru/PIU-005/2022|2022-11-22|PB1|EPI2664209
A/chicken/South_Dakota/22-033852-001-original/2022|2022-10-21|PB1|EPI2263768
A/chicken/Wyoming/22-032216-001-original/2022|2022-10-10|PB1|EPI2264185
A/chicken/Colorado/22-032233-001-original/2022|2022-10-10|PB1|EPI2264161
A/chicken/Colorado/22-032670-001-original/2022|2022-10-10|PB1|EPI2264105
A/crow/Minnesota/23-007350-001-original/2023|2023-03-03|PB1|EPI2613884
A/guinea_fowl/Oklahoma/22-032674-001-original/2022|2022-10-13|PB1|EPI2264113
A/red-tailed_hawk/Kansas/23-009211-001-original/2023|2023-03-15|PB1|EPI2614084
A/great_horned_owl/Wyoming/23-014892-001-original/2023|2023-04-13|PB1|EPI2614484
81
A/Cooper_s_hawk/Colorado/23-005887-001-original/2023|2023-02-18|PB1|EPI2613732
75
A/Cooper_ss_hawk/Wyoming/23-013975-001-original/2023|2023-04-29|PB1|EPI2614412
A/great_horned_owl/Colorado/23-015245-001-original/2023|2023-05-09|PB1|EPI2614548

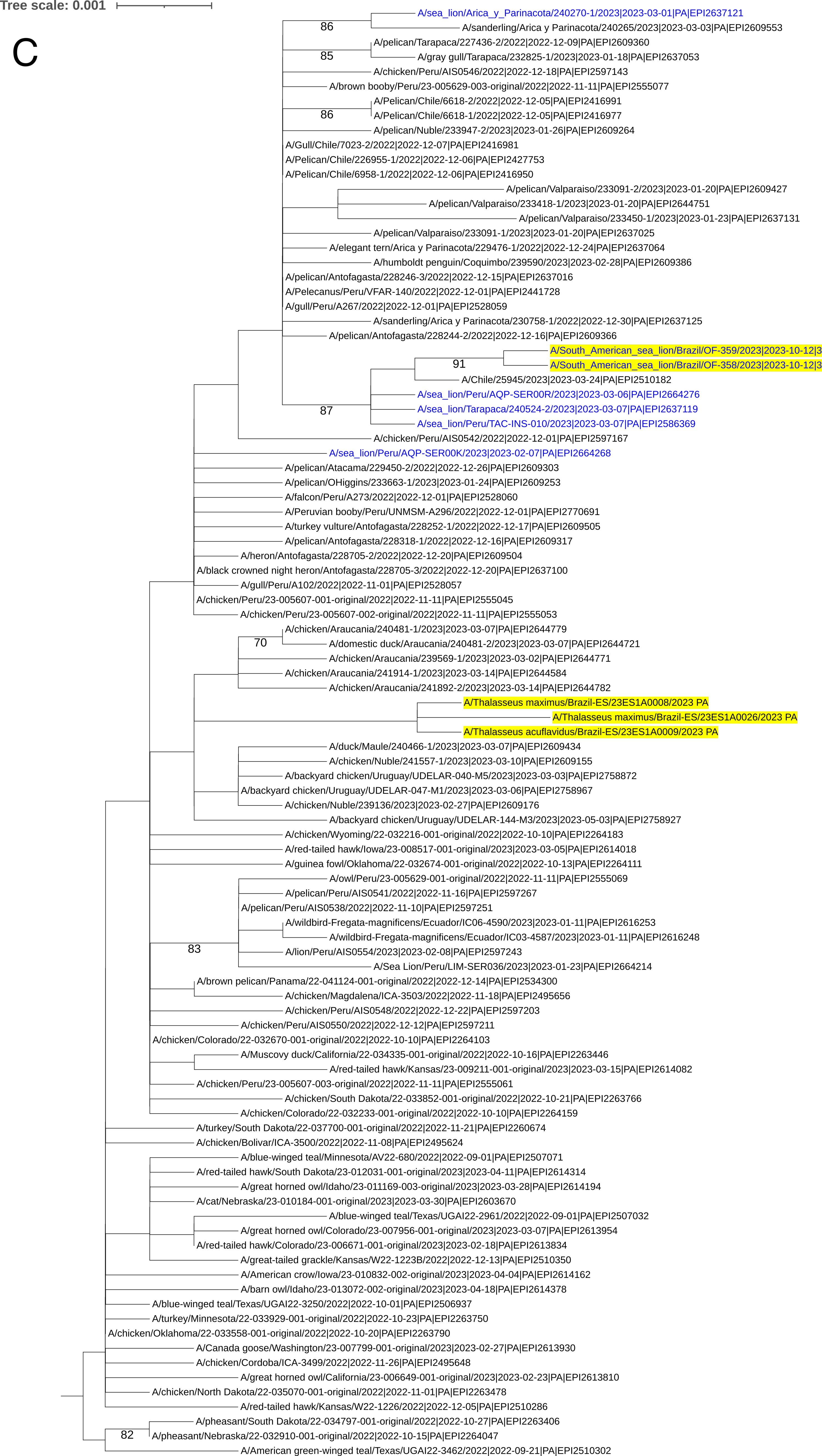
Tree scale: 0.001
C
A/sea_lion/Arica_y_Parinacota/240270-1/2023|2023-03-01|PA|EPI2637121
86
A/sanderling/Arica y Parinacota/240265/2023|2023-03-03|PA|EPI2609553
A/pelican/Tarapaca/227436-2/2022|2022-12-09|PA|EPI2609360
85
A/gray gull/Tarapaca/232825-1/2023|2023-01-18|PA|EPI2637053
A/chicken/Peru/AIS0546/2022|2022-12-18|PA|EPI2597143
A/brown booby/Peru/23-005629-003-original/2022|2022-11-11|PA|EPI2555077
A/Pelican/Chile/6618-2/2022|2022-12-05|PA|EPI2416991
86
A/Pelican/Chile/6618-1/2022|2022-12-05|PA|EPI2416977
A/pelican/Nuble/233947-2/2023|2023-01-26|PA|EPI2609264
A/Gull/Chile/7023-2/2022|2022-12-07|PA|EPI2416981
A/Pelican/Chile/226955-1/2022|2022-12-06|PA|EPI2427753
A/Pelican/Chile/6958-1/2022|2022-12-06|PA|EPI2416950
A/pelican/Valparaiso/233091-2/2023|2023-01-20|PA|EPI2609427
A/pelican/Valparaiso/233418-1/2023|2023-01-20|PA|EPI2644751
A/pelican/Valparaiso/233450-1/2023|2023-01-23|PA|EPI2637131
A/pelican/Valparaiso/233091-1/2023|2023-01-20|PA|EPI2637025
A/elegant tern/Arica y Parinacota/229476-1/2022|2022-12-24|PA|EPI2637064
A/humboldt penguin/Coquimbo/239590/2023|2023-02-28|PA|EPI2609386
A/pelican/Antofagasta/228246-3/2022|2022-12-15|PA|EPI2637016
A/Pelecanus/Peru/VFAR-140/2022|2022-12-01|PA|EPI2441728
A/gull/Peru/A267/2022|2022-12-01|PA|EPI2528059
A/sanderling/Arica y Parinacota/230758-1/2022|2022-12-30|PA|EPI2637125
A/pelican/Antofagasta/228244-2/2022|2022-12-16|PA|EPI2609366
A/South_American_sea_lion/Brazil/OF-359/2023|2023-10-12|3
91
A/South_American_sea_lion/Brazil/OF-358/2023|2023-10-12|3
A/Chile/25945/2023|2023-03-24|PA|EPI2510182
A/sea_lion/Peru/AQP-SER00R/2023|2023-03-06|PA|EPI2664276
87
A/sea_lion/Tarapaca/240524-2/2023|2023-03-07|PA|EPI2637119
A/sea_lion/Peru/TAC-INS-010/2023|2023-03-07|PA|EPI2586369
A/chicken/Peru/AIS0542/2022|2022-12-01|PA|EPI2597167
A/sea_lion/Peru/AQP-SER00K/2023|2023-02-07|PA|EPI2664268
A/pelican/Atacama/229450-2/2022|2022-12-26|PA|EPI2609303
A/pelican/OHiggins/233663-1/2023|2023-01-24|PA|EPI2609253
A/falcon/Peru/A273/2022|2022-12-01|PA|EPI2528060
A/Peruvian booby/Peru/UNMSM-A296/2022|2022-12-01|PA|EPI2770691
A/turkey vulture/Antofagasta/228252-1/2022|2022-12-17|PA|EPI2609505
A/pelican/Antofagasta/228318-1/2022|2022-12-16|PA|EPI2609317
A/heron/Antofagasta/228705-2/2022|2022-12-20|PA|EPI2609504
A/black crowned night heron/Antofagasta/228705-3/2022|2022-12-20|PA|EPI2637100
A/gull/Peru/A102/2022|2022-11-01|PA|EPI2528057
A/chicken/Peru/23-005607-001-original/2022|2022-11-11|PA|EPI2555045
A/chicken/Peru/23-005607-002-original/2022|2022-11-11|PA|EPI2555053
A/chicken/Araucania/240481-1/2023|2023-03-07|PA|EPI2644779
70
A/domestic duck/Araucania/240481-2/2023|2023-03-07|PA|EPI2644721
A/chicken/Araucania/239569-1/2023|2023-03-02|PA|EPI2644771
A/chicken/Araucania/241914-1/2023|2023-03-14|PA|EPI2644584
A/chicken/Araucania/241892-2/2023|2023-03-14|PA|EPI2644782
A/Thalasseus maximus/Brazil-ES/23ES1A0008/2023 PA
A/Thalasseus maximus/Brazil-ES/23ES1A0026/2023 PA
A/Thalasseus acuflavidus/Brazil-ES/23ES1A0009/2023 PA
A/duck/Maule/240466-1/2023|2023-03-07|PA|EPI2609434
A/chicken/Nuble/241557-1/2023|2023-03-10|PA|EPI2609155
A/backyard chicken/Uruguay/UDELAR-040-M5/2023|2023-03-03|PA|EPI2758872
A/backyard chicken/Uruguay/UDELAR-047-M1/2023|2023-03-06|PA|EPI2758967
A/chicken/Nuble/239136/2023|2023-02-27|PA|EPI2609176
A/backyard chicken/Uruguay/UDELAR-144-M3/2023|2023-05-03|PA|EPI2758927
A/chicken/Wyoming/22-032216-001-original/2022|2022-10-10|PA|EPI2264183
A/red-tailed hawk/Iowa/23-008517-001-original/2023|2023-03-05|PA|EPI2614018
A/guinea fowl/Oklahoma/22-032674-001-original/2022|2022-10-13|PA|EPI2264111
A/owl/Peru/23-005629-001-original/2022|2022-11-11|PA|EPI2555069
A/pelican/Peru/AIS0541/2022|2022-11-16|PA|EPI2597267
A/pelican/Peru/AIS0538/2022|2022-11-10|PA|EPI2597251
A/wildbird-Fregata-magnificens/Ecuador/IC06-4590/2023|2023-01-11|PA|EPI2616253
A/wildbird-Fregata-magnificens/Ecuador/IC03-4587/2023|2023-01-11|PA|EPI2616248
83
A/lion/Peru/AIS0554/2023|2023-02-08|PA|EPI2597243
A/Sea Lion/Peru/LIM-SER036/2023|2023-01-23|PA|EPI2664214
A/brown pelican/Panama/22-041124-001-original/2022|2022-12-14|PA|EPI2534300
A/chicken/Magdalena/ICA-3503/2022|2022-11-18|PA|EPI2495656
A/chicken/Peru/AIS0548/2022|2022-12-22|PA|EPI2597203
A/chicken/Peru/AIS0550/2022|2022-12-12|PA|EPI2597211
A/chicken/Colorado/22-032670-001-original/2022|2022-10-10|PA|EPI2264103
A/Muscovy duck/California/22-034335-001-original/2022|2022-10-16|PA|EPI2263446
A/red-tailed hawk/Kansas/23-009211-001-original/2023|2023-03-15|PA|EPI2614082
A/chicken/Peru/23-005607-003-original/2022|2022-11-11|PA|EPI2555061
A/chicken/South Dakota/22-033852-001-original/2022|2022-10-21|PA|EPI2263766
A/chicken/Colorado/22-032233-001-original/2022|2022-10-10|PA|EPI2264159
A/turkey/South Dakota/22-037700-001-original/2022|2022-11-21|PA|EPI2260674
A/chicken/Bolivar/ICA-3500/2022|2022-11-08|PA|EPI2495624
A/blue-winged teal/Minnesota/AV22-680/2022|2022-09-01|PA|EPI2507071
A/red-tailed hawk/South Dakota/23-012031-001-original/2023|2023-04-11|PA|EPI2614314
A/great horned owl/Idaho/23-011169-003-original/2023|2023-03-28|PA|EPI2614194
A/cat/Nebraska/23-010184-001-original/2023|2023-03-30|PA|EPI2603670
A/blue-winged teal/Texas/UGAI22-2961/2022|2022-09-01|PA|EPI2507032
A/great horned owl/Colorado/23-007956-001-original/2023|2023-03-07|PA|EPI2613954
A/red-tailed hawk/Colorado/23-006671-001-original/2023|2023-02-18|PA|EPI2613834
A/great-tailed grackle/Kansas/W22-1223B/2022|2022-12-13|PA|EPI2510350
A/American crow/Iowa/23-010832-002-original/2023|2023-04-04|PA|EPI2614162
A/barn owl/Idaho/23-013072-002-original/2023|2023-04-18|PA|EPI2614378
A/blue-winged teal/Texas/UGAI22-3250/2022|2022-10-01|PA|EPI2506937
A/turkey/Minnesota/22-033929-001-original/2022|2022-10-23|PA|EPI2263750
A/chicken/Oklahoma/22-033558-001-original/2022|2022-10-20|PA|EPI2263790
A/Canada goose/Washington/23-007799-001-original/2023|2023-02-27|PA|EPI2613930
A/chicken/Cordoba/ICA-3499/2022|2022-11-26|PA|EPI2495648
A/great horned owl/California/23-006649-001-original/2023|2023-02-23|PA|EPI2613810
A/chicken/North Dakota/22-035070-001-original/2022|2022-11-01|PA|EPI2263478
A/red-tailed hawk/Kansas/W22-1226/2022|2022-12-05|PA|EPI2510286
A/pheasant/South Dakota/22-034797-001-original/2022|2022-10-27|PA|EPI2263406
82
A/pheasant/Nebraska/22-032910-001-original/2022|2022-10-15|PA|EPI2264047
A/American green-winged teal/Texas/UGAI22-3462/2022|2022-09-21|PA|EPI2510302

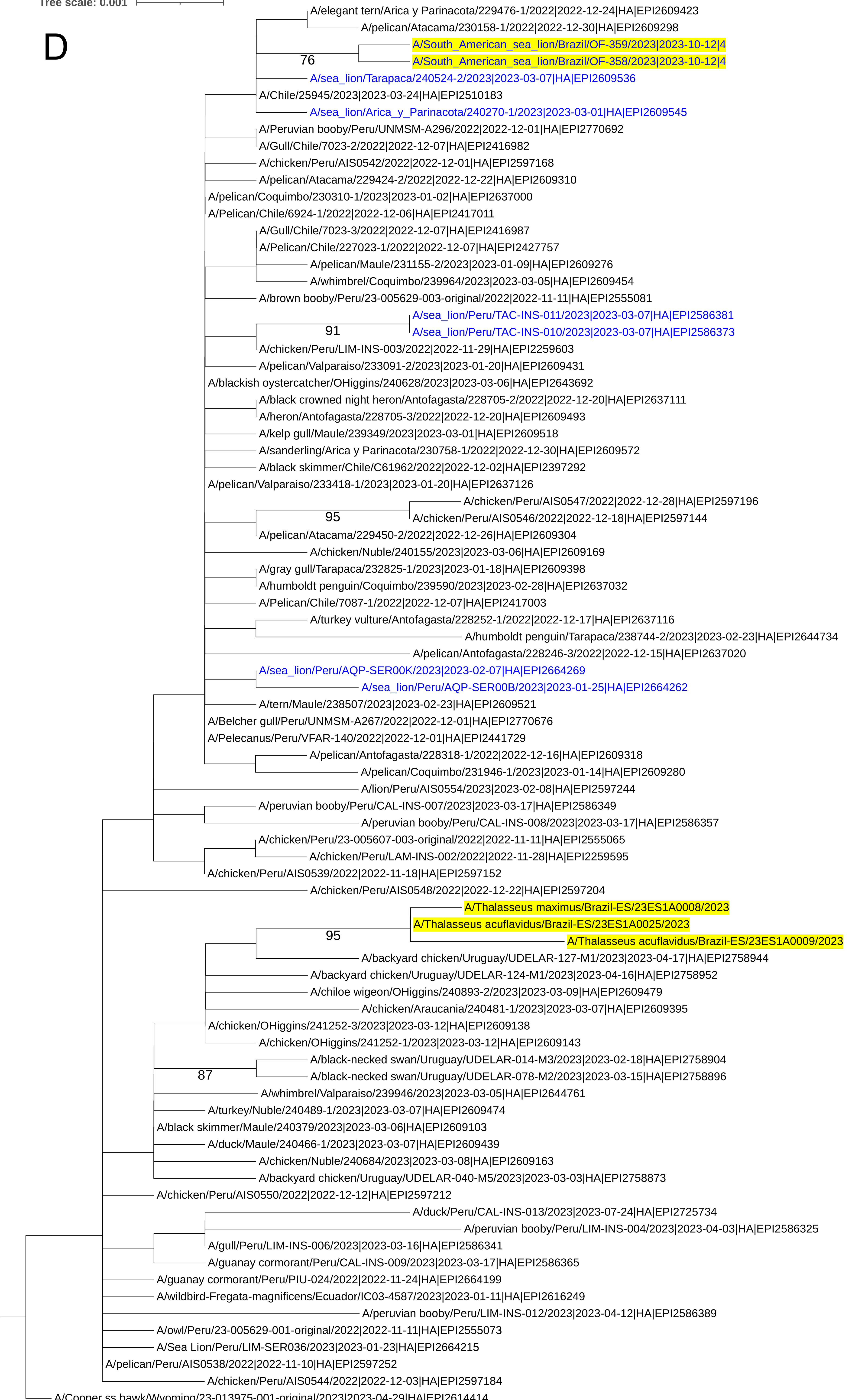
Tree scale: 0.001
D
A/elegant tern/Arica y Parinacota/229476-1/2022|2022-12-24|HA|EPI2609423
A/pelican/Atacama/230158-1/2022|2022-12-30|HA|EPI2609298
A/South_American_sea_lion/Brazil/OF-359/2023|2023-10-12|4
76
A/South_American_sea_lion/Brazil/OF-358/2023|2023-10-12|4
A/sea_lion/Tarapaca/240524-2/2023|2023-03-07|HA|EPI2609536
A/Chile/25945/2023|2023-03-24|HA|EPI2510183
A/sea_lion/Arica_y_Parinacota/240270-1/2023|2023-03-01|HA|EPI2609545
A/Peruvian booby/Peru/UNMSM-A296/2022|2022-12-01|HA|EPI2770692
A/Gull/Chile/7023-2/2022|2022-12-07|HA|EPI2416982
A/chicken/Peru/AIS0542/2022|2022-12-01|HA|EPI2597168
A/pelican/Atacama/229424-2/2022|2022-12-22|HA|EPI2609310
A/pelican/Coquimbo/230310-1/2023|2023-01-02|HA|EPI2637000
A/Pelican/Chile/6924-1/2022|2022-12-06|HA|EPI2417011
A/Gull/Chile/7023-3/2022|2022-12-07|HA|EPI2416987
A/Pelican/Chile/227023-1/2022|2022-12-07|HA|EPI2427757
A/pelican/Maule/231155-2/2023|2023-01-09|HA|EPI2609276
A/whimbrel/Coquimbo/239964/2023|2023-03-05|HA|EPI2609454
A/brown booby/Peru/23-005629-003-original/2022|2022-11-11|HA|EPI2555081
A/sea_lion/Peru/TAC-INS-011/2023|2023-03-07|HA|EPI2586381
91
A/sea_lion/Peru/TAC-INS-010/2023|2023-03-07|HA|EPI2586373
A/chicken/Peru/LIM-INS-003/2022|2022-11-29|HA|EPI2259603
A/pelican/Valparaiso/233091-2/2023|2023-01-20|HA|EPI2609431
A/blackish oystercatcher/OHiggins/240628/2023|2023-03-06|HA|EPI2643692
A/black crowned night heron/Antofagasta/228705-2/2022|2022-12-20|HA|EPI2637111
A/heron/Antofagasta/228705-3/2022|2022-12-20|HA|EPI2609493
A/kelp gull/Maule/239349/2023|2023-03-01|HA|EPI2609518
A/sanderling/Arica y Parinacota/230758-1/2022|2022-12-30|HA|EPI2609572
A/black skimmer/Chile/C61962/2022|2022-12-02|HA|EPI2397292
A/pelican/Valparaiso/233418-1/2023|2023-01-20|HA|EPI2637126
A/chicken/Peru/AIS0547/2022|2022-12-28|HA|EPI2597196
95
A/chicken/Peru/AIS0546/2022|2022-12-18|HA|EPI2597144
A/pelican/Atacama/229450-2/2022|2022-12-26|HA|EPI2609304
A/chicken/Nuble/240155/2023|2023-03-06|HA|EPI2609169
A/gray gull/Tarapaca/232825-1/2023|2023-01-18|HA|EPI2609398
A/humboldt penguin/Coquimbo/239590/2023|2023-02-28|HA|EPI2637032
A/Pelican/Chile/7087-1/2022|2022-12-07|HA|EPI2417003
A/turkey vulture/Antofagasta/228252-1/2022|2022-12-17|HA|EPI2637116
A/humboldt penguin/Tarapaca/238744-2/2023|2023-02-23|HA|EPI2644734
A/pelican/Antofagasta/228246-3/2022|2022-12-15|HA|EPI2637020
A/sea_lion/Peru/AQP-SER00K/2023|2023-02-07|HA|EPI2664269
A/sea_lion/Peru/AQP-SER00B/2023|2023-01-25|HA|EPI2664262
A/tern/Maule/238507/2023|2023-02-23|HA|EPI2609521
A/Belcher gull/Peru/UNMSM-A267/2022|2022-12-01|HA|EPI2770676
A/Pelecanus/Peru/VFAR-140/2022|2022-12-01|HA|EPI2441729
A/pelican/Antofagasta/228318-1/2022|2022-12-16|HA|EPI2609318
A/pelican/Coquimbo/231946-1/2023|2023-01-14|HA|EPI2609280
A/lion/Peru/AIS0554/2023|2023-02-08|HA|EPI2597244
A/peruvian booby/Peru/CAL-INS-007/2023|2023-03-17|HA|EPI2586349
A/peruvian booby/Peru/CAL-INS-008/2023|2023-03-17|HA|EPI2586357
A/chicken/Peru/23-005607-003-original/2022|2022-11-11|HA|EPI2555065
A/chicken/Peru/LAM-INS-002/2022|2022-11-28|HA|EPI2259595
A/chicken/Peru/AIS0539/2022|2022-11-18|HA|EPI2597152
A/chicken/Peru/AIS0548/2022|2022-12-22|HA|EPI2597204
A/Thalasseus maximus/Brazil-ES/23ES1A0008/2023
A/Thalasseus acuflavidus/Brazil-ES/23ES1A0025/2023
95
A/Thalasseus acuflavidus/Brazil-ES/23ES1A0009/2023
A/backyard chicken/Uruguay/UDELAR-127-M1/2023|2023-04-17|HA|EPI2758944
A/backyard chicken/Uruguay/UDELAR-124-M1/2023|2023-04-16|HA|EPI2758952
A/chiloe wigeon/OHiggins/240893-2/2023|2023-03-09|HA|EPI2609479
A/chicken/Araucania/240481-1/2023|2023-03-07|HA|EPI2609395
A/chicken/OHiggins/241252-3/2023|2023-03-12|HA|EPI2609138
A/chicken/OHiggins/241252-1/2023|2023-03-12|HA|EPI2609143
A/black-necked swan/Uruguay/UDELAR-014-M3/2023|2023-02-18|HA|EPI2758904
87
A/black-necked swan/Uruguay/UDELAR-078-M2/2023|2023-03-15|HA|EPI2758896
A/whimbrel/Valparaiso/239946/2023|2023-03-05|HA|EPI2644761
A/turkey/Nuble/240489-1/2023|2023-03-07|HA|EPI2609474
A/black skimmer/Maule/240379/2023|2023-03-06|HA|EPI2609103
A/duck/Maule/240466-1/2023|2023-03-07|HA|EPI2609439
A/chicken/Nuble/240684/2023|2023-03-08|HA|EPI2609163
A/backyard chicken/Uruguay/UDELAR-040-M5/2023|2023-03-03|HA|EPI2758873
A/chicken/Peru/AIS0550/2022|2022-12-12|HA|EPI2597212
A/duck/Peru/CAL-INS-013/2023|2023-07-24|HA|EPI2725734
A/peruvian booby/Peru/LIM-INS-004/2023|2023-04-03|HA|EPI2586325
A/gull/Peru/LIM-INS-006/2023|2023-03-16|HA|EPI2586341
A/guanay cormorant/Peru/CAL-INS-009/2023|2023-03-17|HA|EPI2586365
A/guanay cormorant/Peru/PIU-024/2022|2022-11-24|HA|EPI2664199
A/wildbird-Fregata-magnificens/Ecuador/IC03-4587/2023|2023-01-11|HA|EPI2616249
A/peruvian booby/Peru/LIM-INS-012/2023|2023-04-12|HA|EPI2586389
A/owl/Peru/23-005629-001-original/2022|2022-11-11|HA|EPI2555073
A/Sea Lion/Peru/LIM-SER036/2023|2023-01-23|HA|EPI2664215
A/pelican/Peru/AIS0538/2022|2022-11-10|HA|EPI2597252
A/chicken/Peru/AIS0544/2022|2022-12-03|HA|EPI2597184
A/Cooper ss hawk/Wyoming/23-013975-001-original/2023|2023-04-29|HA|EPI2614414

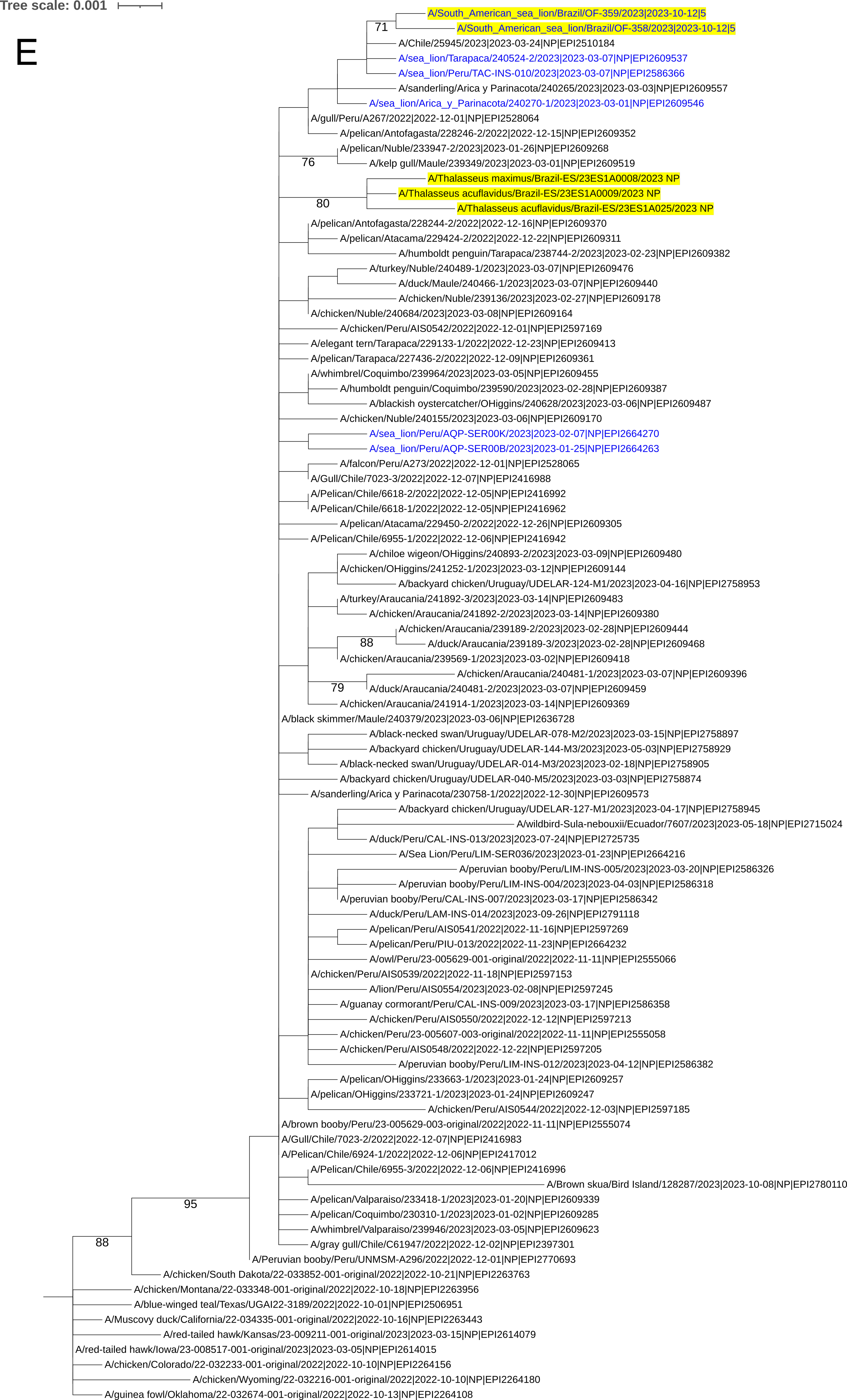
Tree scale: 0.001
E
A/South_American_sea_lion/Brazil/OF-359/2023|2023-10-12|5
71
A/South_American_sea_lion/Brazil/OF-358/2023|2023-10-12|5
A/Chile/25945/2023|2023-03-24|NP|EPI2510184
A/sea_lion/Tarapaca/240524-2/2023|2023-03-07|NP|EPI2609537
A/sea_lion/Peru/TAC-INS-010/2023|2023-03-07|NP|EPI2586366
A/sanderling/Arica y Parinacota/240265/2023|2023-03-03|NP|EPI2609557
A/sea_lion/Arica_y_Parinacota/240270-1/2023|2023-03-01|NP|EPI2609546
A/gull/Peru/A267/2022|2022-12-01|NP|EPI2528064
A/pelican/Antofagasta/228246-2/2022|2022-12-15|NP|EPI2609352
A/pelican/Nuble/233947-2/2023|2023-01-26|NP|EPI2609268
76
A/kelp gull/Maule/239349/2023|2023-03-01|NP|EPI2609519
A/Thalasseus maximus/Brazil-ES/23ES1A0008/2023 NP
A/Thalasseus acuflavidus/Brazil-ES/23ES1A0009/2023 NP
80
A/Thalasseus acuflavidus/Brazil-ES/23ES1A025/2023 NP
A/pelican/Antofagasta/228244-2/2022|2022-12-16|NP|EPI2609370
A/pelican/Atacama/229424-2/2022|2022-12-22|NP|EPI2609311
A/humboldt penguin/Tarapaca/238744-2/2023|2023-02-23|NP|EPI2609382
A/turkey/Nuble/240489-1/2023|2023-03-07|NP|EPI2609476
A/duck/Maule/240466-1/2023|2023-03-07|NP|EPI2609440
A/chicken/Nuble/239136/2023|2023-02-27|NP|EPI2609178
A/chicken/Nuble/240684/2023|2023-03-08|NP|EPI2609164
A/chicken/Peru/AIS0542/2022|2022-12-01|NP|EPI2597169
A/elegant tern/Tarapaca/229133-1/2022|2022-12-23|NP|EPI2609413
A/pelican/Tarapaca/227436-2/2022|2022-12-09|NP|EPI2609361
A/whimbrel/Coquimbo/239964/2023|2023-03-05|NP|EPI2609455
A/humboldt penguin/Coquimbo/239590/2023|2023-02-28|NP|EPI2609387
A/blackish oystercatcher/OHiggins/240628/2023|2023-03-06|NP|EPI2609487
A/chicken/Nuble/240155/2023|2023-03-06|NP|EPI2609170
A/sea_lion/Peru/AQP-SER00K/2023|2023-02-07|NP|EPI2664270
A/sea_lion/Peru/AQP-SER00B/2023|2023-01-25|NP|EPI2664263
A/falcon/Peru/A273/2022|2022-12-01|NP|EPI2528065
A/Gull/Chile/7023-3/2022|2022-12-07|NP|EPI2416988
A/Pelican/Chile/6618-2/2022|2022-12-05|NP|EPI2416992
A/Pelican/Chile/6618-1/2022|2022-12-05|NP|EPI2416962
A/pelican/Atacama/229450-2/2022|2022-12-26|NP|EPI2609305
A/Pelican/Chile/6955-1/2022|2022-12-06|NP|EPI2416942
A/chiloe wigeon/OHiggins/240893-2/2023|2023-03-09|NP|EPI2609480
A/chicken/OHiggins/241252-1/2023|2023-03-12|NP|EPI2609144
A/backyard chicken/Uruguay/UDELAR-124-M1/2023|2023-04-16|NP|EPI2758953
A/turkey/Araucania/241892-3/2023|2023-03-14|NP|EPI2609483
A/chicken/Araucania/241892-2/2023|2023-03-14|NP|EPI2609380
A/chicken/Araucania/239189-2/2023|2023-02-28|NP|EPI2609444
88
A/duck/Araucania/239189-3/2023|2023-02-28|NP|EPI2609468
A/chicken/Araucania/239569-1/2023|2023-03-02|NP|EPI2609418
A/chicken/Araucania/240481-1/2023|2023-03-07|NP|EPI2609396
79
A/duck/Araucania/240481-2/2023|2023-03-07|NP|EPI2609459
A/chicken/Araucania/241914-1/2023|2023-03-14|NP|EPI2609369
A/black skimmer/Maule/240379/2023|2023-03-06|NP|EPI2636728
A/black-necked swan/Uruguay/UDELAR-078-M2/2023|2023-03-15|NP|EPI2758897
A/backyard chicken/Uruguay/UDELAR-144-M3/2023|2023-05-03|NP|EPI2758929
A/black-necked swan/Uruguay/UDELAR-014-M3/2023|2023-02-18|NP|EPI2758905
A/backyard chicken/Uruguay/UDELAR-040-M5/2023|2023-03-03|NP|EPI2758874
A/sanderling/Arica y Parinacota/230758-1/2022|2022-12-30|NP|EPI2609573
A/backyard chicken/Uruguay/UDELAR-127-M1/2023|2023-04-17|NP|EPI2758945
A/wildbird-Sula-nebouxii/Ecuador/7607/2023|2023-05-18|NP|EPI2715024
A/duck/Peru/CAL-INS-013/2023|2023-07-24|NP|EPI2725735
A/Sea Lion/Peru/LIM-SER036/2023|2023-01-23|NP|EPI2664216
A/peruvian booby/Peru/LIM-INS-005/2023|2023-03-20|NP|EPI2586326
A/peruvian booby/Peru/LIM-INS-004/2023|2023-04-03|NP|EPI2586318
A/peruvian booby/Peru/CAL-INS-007/2023|2023-03-17|NP|EPI2586342
A/duck/Peru/LAM-INS-014/2023|2023-09-26|NP|EPI2791118
A/pelican/Peru/AIS0541/2022|2022-11-16|NP|EPI2597269
A/pelican/Peru/PIU-013/2022|2022-11-23|NP|EPI2664232
A/owl/Peru/23-005629-001-original/2022|2022-11-11|NP|EPI2555066
A/chicken/Peru/AIS0539/2022|2022-11-18|NP|EPI2597153
A/lion/Peru/AIS0554/2023|2023-02-08|NP|EPI2597245
A/guanay cormorant/Peru/CAL-INS-009/2023|2023-03-17|NP|EPI2586358
A/chicken/Peru/AIS0550/2022|2022-12-12|NP|EPI2597213
A/chicken/Peru/23-005607-003-original/2022|2022-11-11|NP|EPI2555058
A/chicken/Peru/AIS0548/2022|2022-12-22|NP|EPI2597205
A/peruvian booby/Peru/LIM-INS-012/2023|2023-04-12|NP|EPI2586382
A/pelican/OHiggins/233663-1/2023|2023-01-24|NP|EPI2609257
A/pelican/OHiggins/233721-1/2023|2023-01-24|NP|EPI2609247
A/chicken/Peru/AIS0544/2022|2022-12-03|NP|EPI2597185
A/brown booby/Peru/23-005629-003-original/2022|2022-11-11|NP|EPI2555074
A/Gull/Chile/7023-2/2022|2022-12-07|NP|EPI2416983
A/Pelican/Chile/6924-1/2022|2022-12-06|NP|EPI2417012
A/Pelican/Chile/6955-3/2022|2022-12-06|NP|EPI2416996
A/Brown skua/Bird Island/128287/2023|2023-10-08|NP|EPI2780110
A/pelican/Valparaiso/233418-1/2023|2023-01-20|NP|EPI2609339
95
A/pelican/Coquimbo/230310-1/2023|2023-01-02|NP|EPI2609285
A/whimbrel/Valparaiso/239946/2023|2023-03-05|NP|EPI2609623
88
A/gray gull/Chile/C61947/2022|2022-12-02|NP|EPI2397301
A/Peruvian booby/Peru/UNMSM-A296/2022|2022-12-01|NP|EPI2770693
A/chicken/South Dakota/22-033852-001-original/2022|2022-10-21|NP|EPI2263763
A/chicken/Montana/22-033348-001-original/2022|2022-10-18|NP|EPI2263956
A/blue-winged teal/Texas/UGAI22-3189/2022|2022-10-01|NP|EPI2506951
A/Muscovy duck/California/22-034335-001-original/2022|2022-10-16|NP|EPI2263443
A/red-tailed hawk/Kansas/23-009211-001-original/2023|2023-03-15|NP|EPI2614079
A/red-tailed hawk/Iowa/23-008517-001-original/2023|2023-03-05|NP|EPI2614015
A/chicken/Colorado/22-032233-001-original/2022|2022-10-10|NP|EPI2264156
A/chicken/Wyoming/22-032216-001-original/2022|2022-10-10|NP|EPI2264180
A/guinea fowl/Oklahoma/22-032674-001-original/2022|2022-10-13|NP|EPI2264108

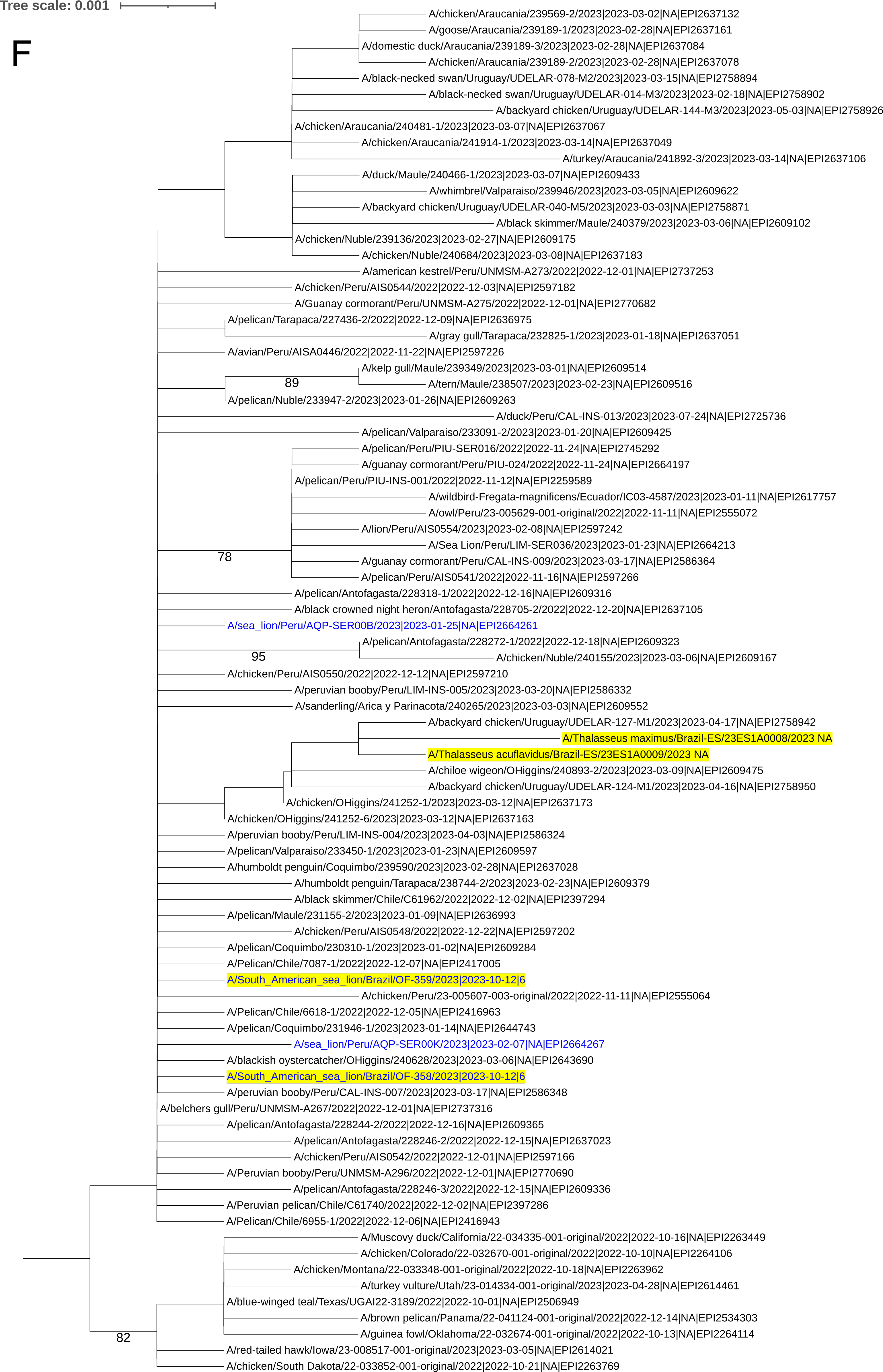

Tree scale: 0.001
F
A/chicken/Araucania/239569-2/2023|2023-03-02|NA|EPI2637132
A/goose/Araucania/239189-1/2023|2023-02-28|NA|EPI2637161
A/domestic duck/Araucania/239189-3/2023|2023-02-28|NA|EPI2637084
A/chicken/Araucania/239189-2/2023|2023-02-28|NA|EPI2637078
A/black-necked swan/Uruguay/UDELAR-078-M2/2023|2023-03-15|NA|EPI2758894
A/black-necked swan/Uruguay/UDELAR-014-M3/2023|2023-02-18|NA|EPI2758902
A/backyard chicken/Uruguay/UDELAR-144-M3/2023|2023-05-03|NA|EPI2758926
A/chicken/Araucania/240481-1/2023|2023-03-07|NA|EPI2637067
A/chicken/Araucania/241914-1/2023|2023-03-14|NA|EPI2637049
A/turkey/Araucania/241892-3/2023|2023-03-14|NA|EPI2637106
A/duck/Maule/240466-1/2023|2023-03-07|NA|EPI2609433
A/whimbrel/Valparaiso/239946/2023|2023-03-05|NA|EPI2609622
A/backyard chicken/Uruguay/UDELAR-040-M5/2023|2023-03-03|NA|EPI2758871
A/black skimmer/Maule/240379/2023|2023-03-06|NA|EPI2609102
A/chicken/Nuble/239136/2023|2023-02-27|NA|EPI2609175
A/chicken/Nuble/240684/2023|2023-03-08|NA|EPI2637183
A/american kestrel/Peru/UNMSM-A273/2022|2022-12-01|NA|EPI2737253
A/chicken/Peru/AIS0544/2022|2022-12-03|NA|EPI2597182
A/Guanay cormorant/Peru/UNMSM-A275/2022|2022-12-01|NA|EPI2770682
A/pelican/Tarapaca/227436-2/2022|2022-12-09|NA|EPI2636975
A/gray gull/Tarapaca/232825-1/2023|2023-01-18|NA|EPI2637051
A/avian/Peru/AISA0446/2022|2022-11-22|NA|EPI2597226
A/kelp gull/Maule/239349/2023|2023-03-01|NA|EPI2609514
89
A/tern/Maule/238507/2023|2023-02-23|NA|EPI2609516
A/pelican/Nuble/233947-2/2023|2023-01-26|NA|EPI2609263
A/duck/Peru/CAL-INS-013/2023|2023-07-24|NA|EPI2725736
A/pelican/Valparaiso/233091-2/2023|2023-01-20|NA|EPI2609425
A/pelican/Peru/PIU-SER016/2022|2022-11-24|NA|EPI2745292
A/guanay cormorant/Peru/PIU-024/2022|2022-11-24|NA|EPI2664197
A/pelican/Peru/PIU-INS-001/2022|2022-11-12|NA|EPI2259589
A/wildbird-Fregata-magnificens/Ecuador/IC03-4587/2023|2023-01-11|NA|EPI2617757
A/owl/Peru/23-005629-001-original/2022|2022-11-11|NA|EPI2555072
A/lion/Peru/AIS0554/2023|2023-02-08|NA|EPI2597242
A/Sea Lion/Peru/LIM-SER036/2023|2023-01-23|NA|EPI2664213
78
A/guanay cormorant/Peru/CAL-INS-009/2023|2023-03-17|NA|EPI2586364
A/pelican/Peru/AIS0541/2022|2022-11-16|NA|EPI2597266
A/pelican/Antofagasta/228318-1/2022|2022-12-16|NA|EPI2609316
A/black crowned night heron/Antofagasta/228705-2/2022|2022-12-20|NA|EPI2637105
A/sea_lion/Peru/AQP-SER00B/2023|2023-01-25|NA|EPI2664261
A/pelican/Antofagasta/228272-1/2022|2022-12-18|NA|EPI2609323
95
A/chicken/Nuble/240155/2023|2023-03-06|NA|EPI2609167
A/chicken/Peru/AIS0550/2022|2022-12-12|NA|EPI2597210
A/peruvian booby/Peru/LIM-INS-005/2023|2023-03-20|NA|EPI2586332
A/sanderling/Arica y Parinacota/240265/2023|2023-03-03|NA|EPI2609552
A/backyard chicken/Uruguay/UDELAR-127-M1/2023|2023-04-17|NA|EPI2758942
A/Thalasseus maximus/Brazil-ES/23ES1A0008/2023 NA
A/Thalasseus acuflavidus/Brazil-ES/23ES1A0009/2023 NA
A/chiloe wigeon/OHiggins/240893-2/2023|2023-03-09|NA|EPI2609475
A/backyard chicken/Uruguay/UDELAR-124-M1/2023|2023-04-16|NA|EPI2758950
A/chicken/OHiggins/241252-1/2023|2023-03-12|NA|EPI2637173
A/chicken/OHiggins/241252-6/2023|2023-03-12|NA|EPI2637163
A/peruvian booby/Peru/LIM-INS-004/2023|2023-04-03|NA|EPI2586324
A/pelican/Valparaiso/233450-1/2023|2023-01-23|NA|EPI2609597
A/humboldt penguin/Coquimbo/239590/2023|2023-02-28|NA|EPI2637028
A/humboldt penguin/Tarapaca/238744-2/2023|2023-02-23|NA|EPI2609379
A/black skimmer/Chile/C61962/2022|2022-12-02|NA|EPI2397294
A/pelican/Maule/231155-2/2023|2023-01-09|NA|EPI2636993
A/chicken/Peru/AIS0548/2022|2022-12-22|NA|EPI2597202
A/pelican/Coquimbo/230310-1/2023|2023-01-02|NA|EPI2609284
A/Pelican/Chile/7087-1/2022|2022-12-07|NA|EPI2417005
A/South_American_sea_lion/Brazil/OF-359/2023|2023-10-12|6
A/chicken/Peru/23-005607-003-original/2022|2022-11-11|NA|EPI2555064
A/Pelican/Chile/6618-1/2022|2022-12-05|NA|EPI2416963
A/pelican/Coquimbo/231946-1/2023|2023-01-14|NA|EPI2644743
A/sea_lion/Peru/AQP-SER00K/2023|2023-02-07|NA|EPI2664267
A/blackish oystercatcher/OHiggins/240628/2023|2023-03-06|NA|EPI2643690
A/South_American_sea_lion/Brazil/OF-358/2023|2023-10-12|6
A/peruvian booby/Peru/CAL-INS-007/2023|2023-03-17|NA|EPI2586348
A/belchers gull/Peru/UNMSM-A267/2022|2022-12-01|NA|EPI2737316
A/pelican/Antofagasta/228244-2/2022|2022-12-16|NA|EPI2609365
A/pelican/Antofagasta/228246-2/2022|2022-12-15|NA|EPI2637023
A/chicken/Peru/AIS0542/2022|2022-12-01|NA|EPI2597166
A/Peruvian booby/Peru/UNMSM-A296/2022|2022-12-01|NA|EPI2770690
A/pelican/Antofagasta/228246-3/2022|2022-12-15|NA|EPI2609336
A/Peruvian pelican/Chile/C61740/2022|2022-12-02|NA|EPI2397286
A/Pelican/Chile/6955-1/2022|2022-12-06|NA|EPI2416943
A/Muscovy duck/California/22-034335-001-original/2022|2022-10-16|NA|EPI2263449
A/chicken/Colorado/22-032670-001-original/2022|2022-10-10|NA|EPI2264106
A/chicken/Montana/22-033348-001-original/2022|2022-10-18|NA|EPI2263962
A/turkey vulture/Utah/23-014334-001-original/2023|2023-04-28|NA|EPI2614461
A/blue-winged teal/Texas/UGAI22-3189/2022|2022-10-01|NA|EPI2506949
A/brown pelican/Panama/22-041124-001-original/2022|2022-12-14|NA|EPI2534303
A/guinea fowl/Oklahoma/22-032674-001-original/2022|2022-10-13|NA|EPI2264114
82
A/red-tailed hawk/Iowa/23-008517-001-original/2023|2023-03-05|NA|EPI2614021
A/chicken/South Dakota/22-033852-001-original/2022|2022-10-21|NA|EPI2263769

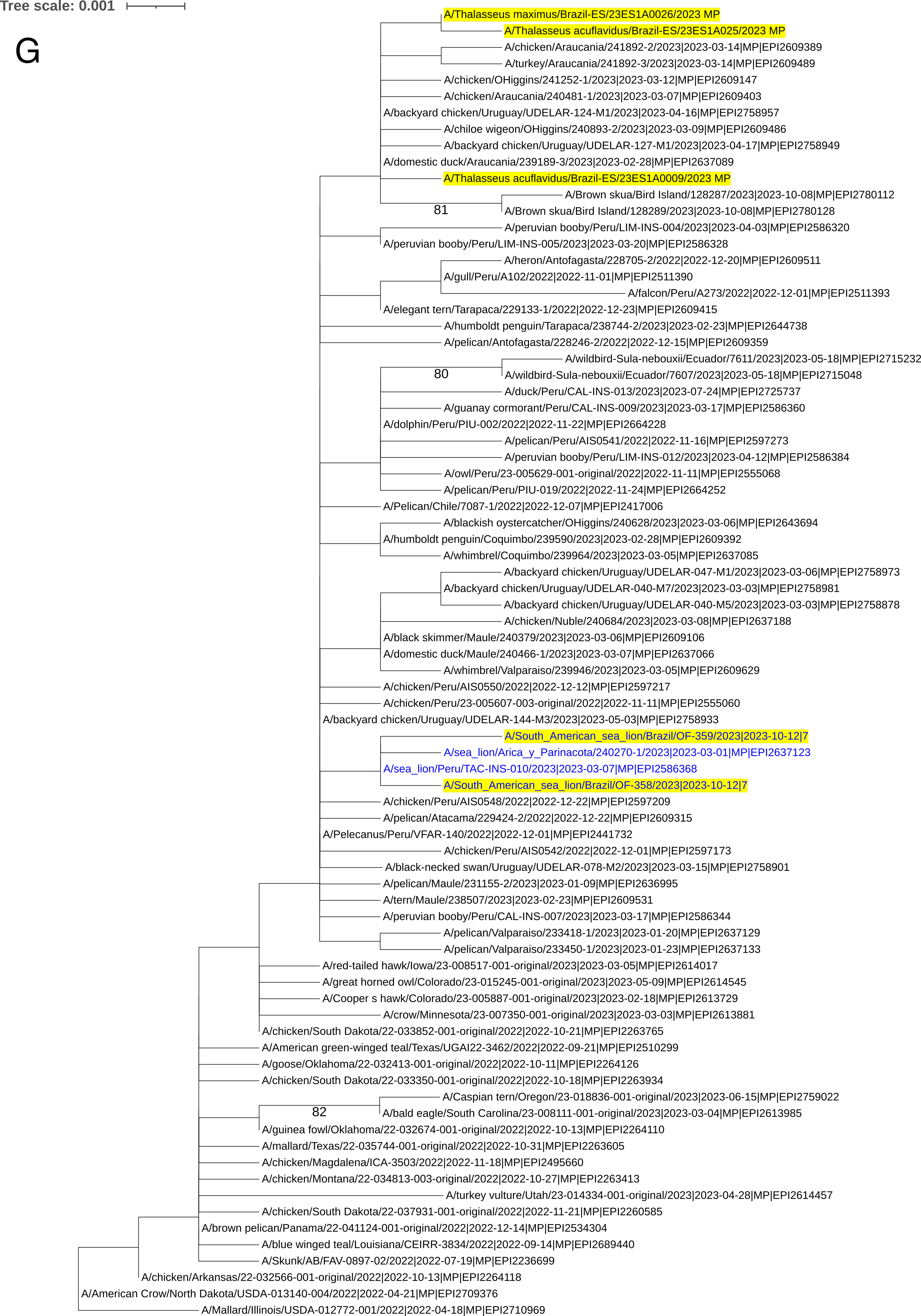
Tree scale: 0.001
G
A/Thalasseus maximus/Brazil-ES/23ES1A0026/2023 MP
A/Thalasseus acuflavidus/Brazil-ES/23ES1A025/2023 MP
A/chicken/Araucania/241892-2/2023|2023-03-14|MP|EPI2609389
A/turkey/Araucania/241892-3/2023|2023-03-14|MP|EPI2609489
A/chicken/OHiggins/241252-1/2023|2023-03-12|MP|EPI2609147
A/chicken/Araucania/240481-1/2023|2023-03-07|MP|EPI2609403
A/backyard chicken/Uruguay/UDELAR-124-M1/2023|2023-04-16|MP|EPI2758957
A/chiloe wigeon/OHiggins/240893-2/2023|2023-03-09|MP|EPI2609486
A/backyard chicken/Uruguay/UDELAR-127-M1/2023|2023-04-17|MP|EPI2758949
A/domestic duck/Araucania/239189-3/2023|2023-02-28|MP|EPI2637089
A/Thalasseus acuflavidus/Brazil-ES/23ES1A0009/2023 MP
A/Brown skua/Bird Island/128287/2023|2023-10-08|MP|EPI2780112
81
A/Brown skua/Bird Island/128289/2023|2023-10-08|MP|EPI2780128
A/peruvian booby/Peru/LIM-INS-004/2023|2023-04-03|MP|EPI2586320
A/peruvian booby/Peru/LIM-INS-005/2023|2023-03-20|MP|EPI2586328
A/heron/Antofagasta/228705-2/2022|2022-12-20|MP|EPI2609511
A/gull/Peru/A102/2022|2022-11-01|MP|EPI2511390
A/falcon/Peru/A273/2022|2022-12-01|MP|EPI2511393
A/elegant tern/Tarapaca/229133-1/2022|2022-12-23|MP|EPI2609415
A/humboldt penguin/Tarapaca/238744-2/2023|2023-02-23|MP|EPI2644738
A/pelican/Antofagasta/228246-2/2022|2022-12-15|MP|EPI2609359
A/wildbird-Sula-nebouxii/Ecuador/7611/2023|2023-05-18|MP|EPI2715232
80
A/wildbird-Sula-nebouxii/Ecuador/7607/2023|2023-05-18|MP|EPI2715048
A/duck/Peru/CAL-INS-013/2023|2023-07-24|MP|EPI2725737
A/guanay cormorant/Peru/CAL-INS-009/2023|2023-03-17|MP|EPI2586360
A/dolphin/Peru/PIU-002/2022|2022-11-22|MP|EPI2664228
A/pelican/Peru/AIS0541/2022|2022-11-16|MP|EPI2597273
A/peruvian booby/Peru/LIM-INS-012/2023|2023-04-12|MP|EPI2586384
A/owl/Peru/23-005629-001-original/2022|2022-11-11|MP|EPI2555068
A/pelican/Peru/PIU-019/2022|2022-11-24|MP|EPI2664252
A/Pelican/Chile/7087-1/2022|2022-12-07|MP|EPI2417006
A/blackish oystercatcher/OHiggins/240628/2023|2023-03-06|MP|EPI2643694
A/humboldt penguin/Coquimbo/239590/2023|2023-02-28|MP|EPI2609392
A/whimbrel/Coquimbo/239964/2023|2023-03-05|MP|EPI2637085
A/backyard chicken/Uruguay/UDELAR-047-M1/2023|2023-03-06|MP|EPI2758973
A/backyard chicken/Uruguay/UDELAR-040-M7/2023|2023-03-03|MP|EPI2758981
A/backyard chicken/Uruguay/UDELAR-040-M5/2023|2023-03-03|MP|EPI2758878
A/chicken/Nuble/240684/2023|2023-03-08|MP|EPI2637188
A/black skimmer/Maule/240379/2023|2023-03-06|MP|EPI2609106
A/domestic duck/Maule/240466-1/2023|2023-03-07|MP|EPI2637066
A/whimbrel/Valparaiso/239946/2023|2023-03-05|MP|EPI2609629
A/chicken/Peru/AIS0550/2022|2022-12-12|MP|EPI2597217
A/chicken/Peru/23-005607-003-original/2022|2022-11-11|MP|EPI2555060
A/backyard chicken/Uruguay/UDELAR-144-M3/2023|2023-05-03|MP|EPI2758933
A/South_American_sea_lion/Brazil/OF-359/2023|2023-10-12|7
A/sea_lion/Arica_y_Parinacota/240270-1/2023|2023-03-01|MP|EPI2637123
A/sea_lion/Peru/TAC-INS-010/2023|2023-03-07|MP|EPI2586368
A/South_American_sea_lion/Brazil/OF-358/2023|2023-10-12|7
A/chicken/Peru/AIS0548/2022|2022-12-22|MP|EPI2597209
A/pelican/Atacama/229424-2/2022|2022-12-22|MP|EPI2609315
A/Pelecanus/Peru/VFAR-140/2022|2022-12-01|MP|EPI2441732
A/chicken/Peru/AIS0542/2022|2022-12-01|MP|EPI2597173
A/black-necked swan/Uruguay/UDELAR-078-M2/2023|2023-03-15|MP|EPI2758901
A/pelican/Maule/231155-2/2023|2023-01-09|MP|EPI2636995
A/tern/Maule/238507/2023|2023-02-23|MP|EPI2609531
A/peruvian booby/Peru/CAL-INS-007/2023|2023-03-17|MP|EPI2586344
A/pelican/Valparaiso/233418-1/2023|2023-01-20|MP|EPI2637129
A/pelican/Valparaiso/233450-1/2023|2023-01-23|MP|EPI2637133
A/red-tailed hawk/Iowa/23-008517-001-original/2023|2023-03-05|MP|EPI2614017
A/great horned owl/Colorado/23-015245-001-original/2023|2023-05-09|MP|EPI2614545
A/Cooper s hawk/Colorado/23-005887-001-original/2023|2023-02-18|MP|EPI2613729
A/crow/Minnesota/23-007350-001-original/2023|2023-03-03|MP|EPI2613881
A/chicken/South Dakota/22-033852-001-original/2022|2022-10-21|MP|EPI2263765
A/American green-winged teal/Texas/UGAI22-3462/2022|2022-09-21|MP|EPI2510299
A/goose/Oklahoma/22-032413-001-original/2022|2022-10-11|MP|EPI2264126
A/chicken/South Dakota/22-033350-001-original/2022|2022-10-18|MP|EPI2263934
A/Caspian tern/Oregon/23-018836-001-original/2023|2023-06-15|MP|EPI2759022
82
A/bald eagle/South Carolina/23-008111-001-original/2023|2023-03-04|MP|EPI2613985
A/guinea fowl/Oklahoma/22-032674-001-original/2022|2022-10-13|MP|EPI2264110
A/mallard/Texas/22-035744-001-original/2022|2022-10-31|MP|EPI2263605
A/chicken/Magdalena/ICA-3503/2022|2022-11-18|MP|EPI2495660
A/chicken/Montana/22-034813-003-original/2022|2022-10-27|MP|EPI2263413
A/turkey vulture/Utah/23-014334-001-original/2023|2023-04-28|MP|EPI2614457
A/chicken/South Dakota/22-037931-001-original/2022|2022-11-21|MP|EPI2260585
A/brown pelican/Panama/22-041124-001-original/2022|2022-12-14|MP|EPI2534304
A/blue winged teal/Louisiana/CEIRR-3834/2022|2022-09-14|MP|EPI2689440
A/Skunk/AB/FAV-0897-02/2022|2022-07-19|MP|EPI2236699
A/chicken/Arkansas/22-032566-001-original/2022|2022-10-13|MP|EPI2264118
A/American Crow/North Dakota/USDA-013140-004/2022|2022-04-21|MP|EPI2709376
A/Mallard/Illinois/USDA-012772-001/2022|2022-04-18|MP|EPI2710969

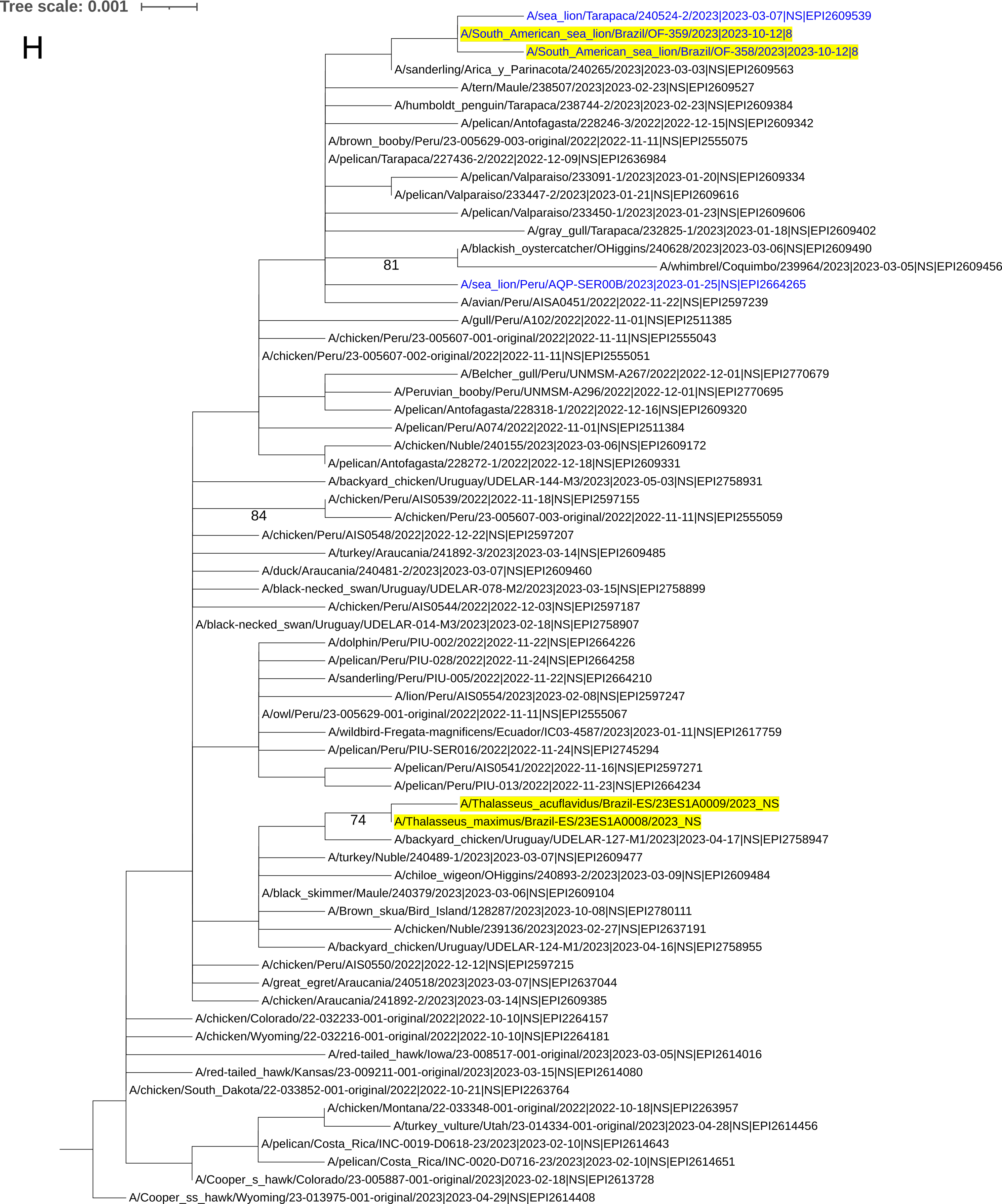
Tree scale: 0.001
H
A/sea_lion/Tarapaca/240524-2/2023|2023-03-07|NS|EPI2609539
A/South_American_sea_lion/Brazil/OF-359/2023|2023-10-12|8
A/South_American_sea_lion/Brazil/OF-358/2023|2023-10-12|8
A/sanderling/Arica_y_Parinacota/240265/2023|2023-03-03|NS|EPI2609563
A/tern/Maule/238507/2023|2023-02-23|NS|EPI2609527
A/humboldt_penguin/Tarapaca/238744-2/2023|2023-02-23|NS|EPI2609384
A/pelican/Antofagasta/228246-3/2022|2022-12-15|NS|EPI2609342
A/brown_booby/Peru/23-005629-003-original/2022|2022-11-11|NS|EPI2555075
A/pelican/Tarapaca/227436-2/2022|2022-12-09|NS|EPI2636984
A/pelican/Valparaiso/233091-1/2023|2023-01-20|NS|EPI2609334
A/pelican/Valparaiso/233447-2/2023|2023-01-21|NS|EPI2609616
A/pelican/Valparaiso/233450-1/2023|2023-01-23|NS|EPI2609606
A/gray_gull/Tarapaca/232825-1/2023|2023-01-18|NS|EPI2609402
A/blackish_oystercatcher/OHiggins/240628/2023|2023-03-06|NS|EPI2609490
81
A/whimbrel/Coquimbo/239964/2023|2023-03-05|NS|EPI2609456
A/sea_lion/Peru/AQP-SER00B/2023|2023-01-25|NS|EPI2664265
A/avian/Peru/AISA0451/2022|2022-11-22|NS|EPI2597239
A/gull/Peru/A102/2022|2022-11-01|NS|EPI2511385
A/chicken/Peru/23-005607-001-original/2022|2022-11-11|NS|EPI2555043
A/chicken/Peru/23-005607-002-original/2022|2022-11-11|NS|EPI2555051
A/Belcher_gull/Peru/UNMSM-A267/2022|2022-12-01|NS|EPI2770679
A/Peruvian_booby/Peru/UNMSM-A296/2022|2022-12-01|NS|EPI2770695
A/pelican/Antofagasta/228318-1/2022|2022-12-16|NS|EPI2609320
A/pelican/Peru/A074/2022|2022-11-01|NS|EPI2511384
A/chicken/Nuble/240155/2023|2023-03-06|NS|EPI2609172
A/pelican/Antofagasta/228272-1/2022|2022-12-18|NS|EPI2609331
A/backyard_chicken/Uruguay/UDELAR-144-M3/2023|2023-05-03|NS|EPI2758931
A/chicken/Peru/AIS0539/2022|2022-11-18|NS|EPI2597155
84
A/chicken/Peru/23-005607-003-original/2022|2022-11-11|NS|EPI2555059
A/chicken/Peru/AIS0548/2022|2022-12-22|NS|EPI2597207
A/turkey/Araucania/241892-3/2023|2023-03-14|NS|EPI2609485
A/duck/Araucania/240481-2/2023|2023-03-07|NS|EPI2609460
A/black-necked_swan/Uruguay/UDELAR-078-M2/2023|2023-03-15|NS|EPI2758899
A/chicken/Peru/AIS0544/2022|2022-12-03|NS|EPI2597187
A/black-necked_swan/Uruguay/UDELAR-014-M3/2023|2023-02-18|NS|EPI2758907
A/dolphin/Peru/PIU-002/2022|2022-11-22|NS|EPI2664226
A/pelican/Peru/PIU-028/2022|2022-11-24|NS|EPI2664258
A/sanderling/Peru/PIU-005/2022|2022-11-22|NS|EPI2664210
A/lion/Peru/AIS0554/2023|2023-02-08|NS|EPI2597247
A/owl/Peru/23-005629-001-original/2022|2022-11-11|NS|EPI2555067
A/wildbird-Fregata-magnificens/Ecuador/IC03-4587/2023|2023-01-11|NS|EPI2617759
A/pelican/Peru/PIU-SER016/2022|2022-11-24|NS|EPI2745294
A/pelican/Peru/AIS0541/2022|2022-11-16|NS|EPI2597271
A/pelican/Peru/PIU-013/2022|2022-11-23|NS|EPI2664234
A/Thalasseus_acuflavidus/Brazil-ES/23ES1A0009/2023_NS
74
A/Thalasseus_maximus/Brazil-ES/23ES1A0008/2023_NS
A/backyard_chicken/Uruguay/UDELAR-127-M1/2023|2023-04-17|NS|EPI2758947
A/turkey/Nuble/240489-1/2023|2023-03-07|NS|EPI2609477
A/chiloe_wigeon/OHiggins/240893-2/2023|2023-03-09|NS|EPI2609484
A/black_skimmer/Maule/240379/2023|2023-03-06|NS|EPI2609104
A/Brown_skua/Bird_Island/128287/2023|2023-10-08|NS|EPI2780111
A/chicken/Nuble/239136/2023|2023-02-27|NS|EPI2637191
A/backyard_chicken/Uruguay/UDELAR-124-M1/2023|2023-04-16|NS|EPI2758955
A/chicken/Peru/AIS0550/2022|2022-12-12|NS|EPI2597215
A/great_egret/Araucania/240518/2023|2023-03-07|NS|EPI2637044
A/chicken/Araucania/241892-2/2023|2023-03-14|NS|EPI2609385
A/chicken/Colorado/22-032233-001-original/2022|2022-10-10|NS|EPI2264157
A/chicken/Wyoming/22-032216-001-original/2022|2022-10-10|NS|EPI2264181
A/red-tailed_hawk/Iowa/23-008517-001-original/2023|2023-03-05|NS|EPI2614016
A/red-tailed_hawk/Kansas/23-009211-001-original/2023|2023-03-15|NS|EPI2614080
A/chicken/South_Dakota/22-033852-001-original/2022|2022-10-21|NS|EPI2263764
A/chicken/Montana/22-033348-001-original/2022|2022-10-18|NS|EPI2263957
A/turkey_vulture/Utah/23-014334-001-original/2023|2023-04-28|NS|EPI2614456
A/pelican/Costa_Rica/INC-0019-D0618-23/2023|2023-02-10|NS|EPI2614643
A/pelican/Costa_Rica/INC-0020-D0716-23/2023|2023-02-10|NS|EPI2614651
A/Cooper_s_hawk/Colorado/23-005887-001-original/2023|2023-02-18|NS|EPI2613728
A/Cooper_ss_hawk/Wyoming/23-013975-001-original/2023|2023-04-29|NS|EPI2614408
