# Supplementary material for: Mortality in sea lions is associated with the introduction of the H5N1 clade 2.3.4.4b virus in Brazil October 2023: whole genome sequencing and phylogenetic analysis
Source: BMC Vet Res. 2024 Jul 2;20:285. doi: 10.1186/s12917-024-04137-1 (PMC11221036; doi:10.1186/s12917-024-04137-1)
Supplement: Supplementary file 2 — Supplementary Material 2: Supplementary Figure 2. Phylogeography of South American H5N1 HPAI using the concatenated sequences of all influenza genome segment. The horizontal axis defines the time scale in decimal years. Branch colors represent different countries as shown in figure legend (AR: Argentina, BO: Bolivar, BR: Brazil, CH: Chile, CO: Columbia, EC: Ecuador, PR: Peru, and UR: Uruguay). [file 12917_2024_4137_MOESM2_ESM.pdf]

Supplementary Material

Supplementary Material was uploaded separately on submission.

Journal: BMC Veterinary Research

Title: Mortality in Sea Lions is associated with the introduction of the H5N1 clade 2.3.4.4b virus in Brazil, October 2023: Whole genome sequencing and phylogenetic analysis

Authors: Andreina Carvalho de Araújo, Andrew Yong Cho, Laura Morais Nascimento Silva, Thais Camilo Corrêa, Gabriela Cristini de Souza, Adriana Silva Albuquerque, Eduardo Macagnan, Cristiane K. M. Kolesnikvoas, Rafael Meurer, Jenyffer Vierheller Vieira, Giulia Gaglianone Lemos, André Silva Barreto, Jeferson Luis Dick, Karina Rejane Groch, Pedro Volkmer de Castilho, Deyvid Amgarten, Fernanda Malta, Michael Miller, Erick G. Dorlass, Soledad Palameta, Sun-Hak Lee, Clarice Weis Arns, Edison L. Durigon, João Renato R. Pinho, Dong-Hun Lee, Helena Lage Ferreira

Supplementary Figure 2. Phylogeography of South American H5N1 HPAI using the concatenated sequences of all influenza genome segment. The horizontal axis defines the time scale in decimal years. Branch colors represent different countries as shown in figure legend (AR: Argentina, BO: Bolivar, BR: Brazil, CH: Chile, CO: Columbia, EC: Ecuador, PR: Peru, and UR: Uruguay).

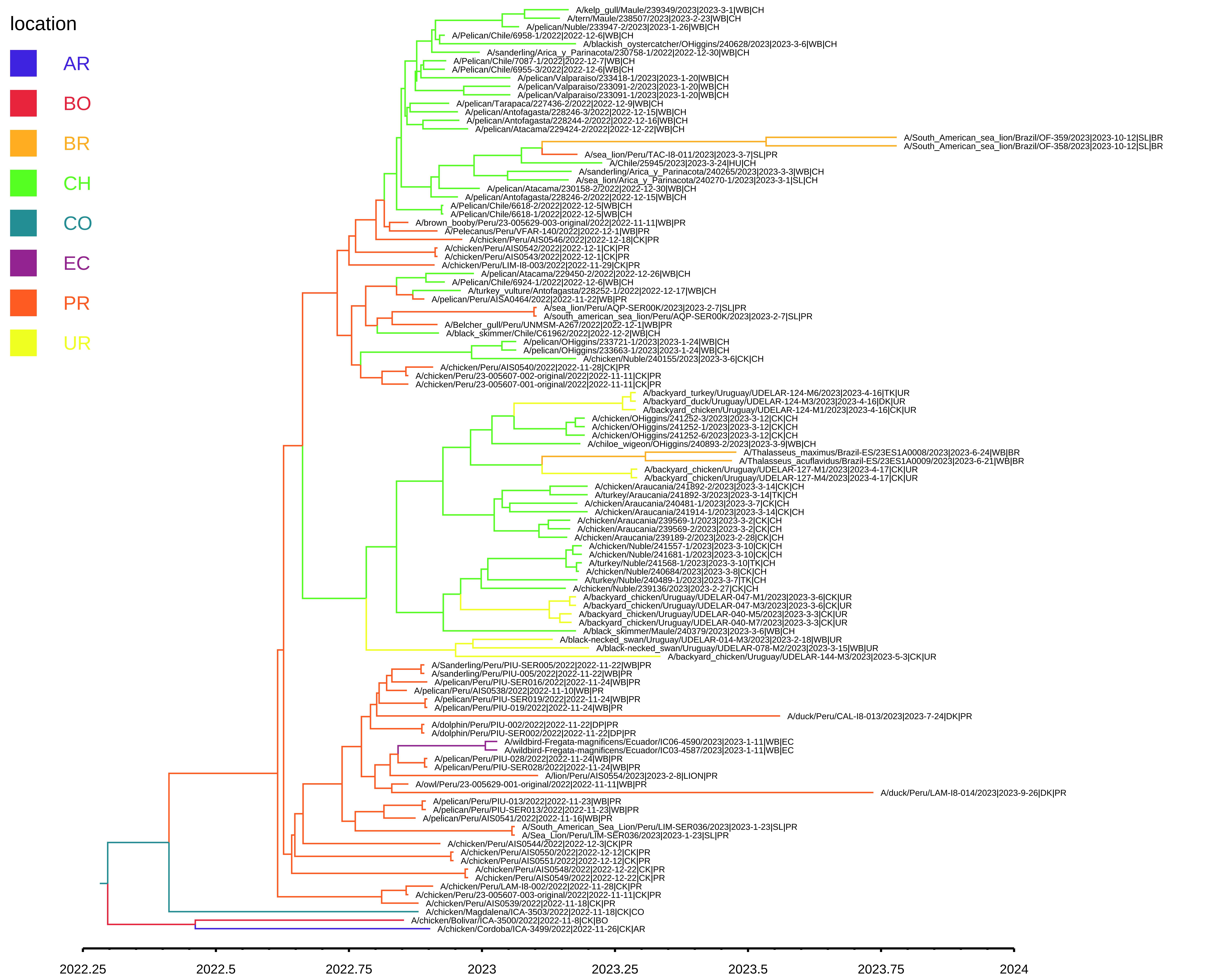
location
AR
BO
BR
CH
CO
EC
PR
UR
A/kelp_gull/Maule/239349/2023|2023-3-1|WB|CH
A/tern/Maule/238507/2023|2023-2-23|WB|CH
A/pelican/Nuble/233947-2/2023|2023-1-26|WB|CH
A/Pelican/Chile/6958-1/2022|2022-12-6|WB|CH
A/blackish_oystercatcher/OHiggins/240628/2023|2023-3-6|WB|CH
A/sanderling/Arica_y_Parinacota/230758-1/2022|2022-12-30|WB|CH
A/Pelican/Chile/7087-1/2022|2022-12-7|WB|CH
A/Pelican/Chile/6955-3/2022|2022-12-6|WB|CH
A/pelican/Valparaiso/233418-1/2023|2023-1-20|WB|CH
A/pelican/Valparaiso/233091-2/2023|2023-1-20|WB|CH
A/pelican/Valparaiso/233091-1/2023|2023-1-20|WB|CH
A/pelican/Tarapaca/227436-2/2022|2022-12-9|WB|CH
A/pelican/Antofagasta/228246-3/2022|2022-12-15|WB|CH
A/pelican/Antofagasta/228244-2/2022|2022-12-16|WB|CH
A/pelican/Atacama/229424-2/2022|2022-12-22|WB|CH
A/South_American_sea_lion/Brazil/OF-359/2023|2023-10-12|SL|BR
A/South_American_sea_lion/Brazil/OF-358/2023|2023-10-12|SL|BR
A/sea_lion/Peru/TAC-I8-011/2023|2023-3-7|SL|PR
A/Chile/25945/2023|2023-3-24|HU|CH
A/sanderling/Arica_y_Parinacota/240265/2023|2023-3-3|WB|CH
A/sea_lion/Arica_y_Parinacota/240270-1/2023|2023-3-1|SL|CH
A/pelican/Atacama/230158-2/2022|2022-12-30|WB|CH
A/pelican/Antofagasta/228246-2/2022|2022-12-15|WB|CH
A/Pelican/Chile/6618-2/2022|2022-12-5|WB|CH
A/Pelican/Chile/6618-1/2022|2022-12-5|WB|CH
A/brown_booby/Peru/23-005629-003-original/2022|2022-11-11|WB|PR
A/Pelecanus/Peru/VFAR-140/2022|2022-12-1|WB|PR
A/chicken/Peru/AIS0546/2022|2022-12-18|CK|PR
A/chicken/Peru/AIS0542/2022|2022-12-1|CK|PR
A/chicken/Peru/AIS0543/2022|2022-12-1|CK|PR
A/chicken/Peru/LIM-I8-003/2022|2022-11-29|CK|PR
A/pelican/Atacama/229450-2/2022|2022-12-26|WB|CH
A/Pelican/Chile/6924-1/2022|2022-12-6|WB|CH
A/turkey_vulture/Antofagasta/228252-1/2022|2022-12-17|WB|CH
A/pelican/Peru/AISA0464/2022|2022-11-22|WB|PR
A/sea_lion/Peru/AQP-SER00K/2023|2023-2-7|SL|PR
A/south_american_sea_lion/Peru/AQP-SER00K/2023|2023-2-7|SL|PR
A/Belcher_gull/Peru/UNMSM-A267/2022|2022-12-1|WB|PR
A/black_skimmer/Chile/C61962/2022|2022-12-2|WB|CH
A/pelican/OHiggins/233721-1/2023|2023-1-24|WB|CH
A/pelican/OHiggins/233663-1/2023|2023-1-24|WB|CH
A/chicken/Nuble/240155/2023|2023-3-6|CK|CH
A/chicken/Peru/AIS0540/2022|2022-11-28|CK|PR
A/chicken/Peru/23-005607-002-original/2022|2022-11-11|CK|PR
A/chicken/Peru/23-005607-001-original/2022|2022-11-11|CK|PR
A/backyard_turkey/Uruguay/UDELAR-124-M6/2023|2023-4-16|TK|UR
A/backyard_duck/Uruguay/UDELAR-124-M3/2023|2023-4-16|DK|UR
A/backyard_chicken/Uruguay/UDELAR-124-M1/2023|2023-4-16|CK|UR
A/chicken/OHiggins/241252-3/2023|2023-3-12|CK|CH
A/chicken/OHiggins/241252-1/2023|2023-3-12|CK|CH
A/chicken/OHiggins/241252-6/2023|2023-3-12|CK|CH
A/chiloe_wigeon/OHiggins/240893-2/2023|2023-3-9|WB|CH
A/Thalasseus_maximus/Brazil-ES/23ES1A0008/2023|2023-6-24|WB|BR
A/Thalasseus_acuflavidus/Brazil-ES/23ES1A0009/2023|2023-6-21|WB|BR
A/backyard_chicken/Uruguay/UDELAR-127-M1/2023|2023-4-17|CK|UR
A/backyard_chicken/Uruguay/UDELAR-127-M4/2023|2023-4-17|CK|UR
A/chicken/Araucania/241892-2/2023|2023-3-14|CK|CH
A/turkey/Araucania/241892-3/2023|2023-3-14|TK|CH
A/chicken/Araucania/240481-1/2023|2023-3-7|CK|CH
A/chicken/Araucania/241914-1/2023|2023-3-14|CK|CH
A/chicken/Araucania/239569-1/2023|2023-3-2|CK|CH
A/chicken/Araucania/239569-2/2023|2023-3-2|CK|CH
A/chicken/Araucania/239189-2/2023|2023-2-28|CK|CH
A/chicken/Nuble/241557-1/2023|2023-3-10|CK|CH
A/chicken/Nuble/241681-1/2023|2023-3-10|CK|CH
A/turkey/Nuble/241568-1/2023|2023-3-10|TK|CH
A/chicken/Nuble/240684/2023|2023-3-8|CK|CH
A/turkey/Nuble/240489-1/2023|2023-3-7|TK|CH
A/chicken/Nuble/239136/2023|2023-2-27|CK|CH
A/backyard_chicken/Uruguay/UDELAR-047-M1/2023|2023-3-6|CK|UR
A/backyard_chicken/Uruguay/UDELAR-047-M3/2023|2023-3-6|CK|UR
A/backyard_chicken/Uruguay/UDELAR-040-M5/2023|2023-3-3|CK|UR
A/backyard_chicken/Uruguay/UDELAR-040-M7/2023|2023-3-3|CK|UR
A/black_skimmer/Maule/240379/2023|2023-3-6|WB|CH
A/black-necked_swan/Uruguay/UDELAR-014-M3/2023|2023-2-18|WB|UR
A/black-necked_swan/Uruguay/UDELAR-078-M2/2023|2023-3-15|WB|UR
A/backyard_chicken/Uruguay/UDELAR-144-M3/2023|2023-5-3|CK|UR
A/Sanderling/Peru/PIU-SER005/2022|2022-11-22|WB|PR
A/sanderling/Peru/PIU-005/2022|2022-11-22|WB|PR
A/pelican/Peru/PIU-SER016/2022|2022-11-24|WB|PR
A/pelican/Peru/AIS0538/2022|2022-11-10|WB|PR
A/pelican/Peru/PIU-SER019/2022|2022-11-24|WB|PR
A/pelican/Peru/PIU-019/2022|2022-11-24|WB|PR
A/duck/Peru/CAL-I8-013/2023|2023-7-24|DK|PR
A/dolphin/Peru/PIU-002/2022|2022-11-22|DP|PR
A/dolphin/Peru/PIU-SER002/2022|2022-11-22|DP|PR
A/wildbird-Fregata-magnificens/Ecuador/IC06-4590/2023|2023-1-11|WB|EC
A/wildbird-Fregata-magnificens/Ecuador/IC03-4587/2023|2023-1-11|WB|EC
A/pelican/Peru/PIU-028/2022|2022-11-24|WB|PR
A/pelican/Peru/PIU-SER028/2022|2022-11-24|WB|PR
A/lion/Peru/AIS0554/2023|2023-2-8|LION|PR
A/owl/Peru/23-005629-001-original/2022|2022-11-11|WB|PR
A/duck/Peru/LAM-I8-014/2023|2023-9-26|DK|PR
A/pelican/Peru/PIU-013/2022|2022-11-23|WB|PR
A/pelican/Peru/PIU-SER013/2022|2022-11-23|WB|PR
A/pelican/Peru/AIS0541/2022|2022-11-16|WB|PR
A/South_American_Sea_Lion/Peru/LIM-SER036/2023|2023-1-23|SL|PR
A/Sea_Lion/Peru/LIM-SER036/2023|2023-1-23|SL|PR
A/chicken/Peru/AIS0544/2022|2022-12-3|CK|PR
A/chicken/Peru/AIS0550/2022|2022-12-12|CK|PR
A/chicken/Peru/AIS0551/2022|2022-12-12|CK|PR
A/chicken/Peru/AIS0548/2022|2022-12-22|CK|PR
A/chicken/Peru/AIS0549/2022|2022-12-22|CK|PR
A/chicken/Peru/LAM-I8-002/2022|2022-11-28|CK|PR
A/chicken/Peru/23-005607-003-original/2022|2022-11-11|CK|PR
A/chicken/Peru/AIS0539/2022|2022-11-18|CK|PR
A/chicken/Magdalena/ICA-3503/2022|2022-11-18|CK|CO
A/chicken/Bolivar/ICA-3500/2022|2022-11-8|CK|BO
A/chicken/Cordoba/ICA-3499/2022|2022-11-26|CK|AR
2022.25
2022.5
2022.75
2023
2023.25
2023.5
2023.75
2024
